# Sustainable solutions for water scarcity: a review of electrostatic fog harvesting technology

**DOI:** 10.1038/s44172-025-00381-x

**Published:** 2025-02-26

**Authors:** Dingchen Li, Chuan Li, Menghan Xiao, Ming Zhang, Jiawei Li, Zhiwen Yang, Qixiong Fu, Kexun Yu, Yong Yang, Yuan Pan, Yaping Du, Xiangen Zhao

**Affiliations:** 1 State Key Laboratory of Advanced Electromagnetic Engineering and Technology, School of Electrical Engineering and Electronics, Huazhong University of Science and Technology, Wuhan, China; 2 Department of Building Environment and Energy Engineering, The Hong Kong Polytechnic University, Hong Kong, China

**Keywords:** Nanoscale materials, Electronic materials

## Abstract

Amid global climate change and population growth, traditional water acquisition methods face challenges. Electrostatic fog harvesting technology offers a novel solution for arid regions, leveraging space charges and electric fields to convert fog into usable water. This article explores the fundamental processes, structure, and enhancement methods of electrostatic fog collectors (EFC), focusing on recent research progress. We offer a prospective perspective on the future research of electrostatic fog harvesting technology, with the aim of facilitating the transition of this technology from scientific research to practical application.

## Abstract (web summary)

Unconventional water resources are becoming increasingly important particularly in waterstressed regions. This review highlights the fundamental physical processes and improvement strategies of atmospheric water harvesting using electrostatic technology, and provides insights into future research directions towards practical applications.

## Introduction

Global water scarcity has emerged as one of the most pressing challenges for human survival and social development in the present century^1^. With the continual growth of the population, the rapid advancement of industrialization and urbanization, and the uncertainties induced by climate change, the contradiction between the supply and demand of water resources is intensifying^2^. According to the latest reports issued by UNESCO (United Nations Educational, Scientific and Cultural Organization) and the United Nations Water Mechanism, approximately 2 ~ 3 billion people worldwide are currently confronting water shortages, and it is expected that the global water demand will increase by 50% by the middle of this century^3,4^. Hence, expanding the channels for water resource acquisition is of exceptional significance for future human responses to the water crisis and the achievement of the United Nations Sustainable Development Goals (UNSDGs).

Water resources, categorized based on their distribution characteristics, can generally be divided into three types: surface water, groundwater, and atmospheric water^5,6^. Currently, humans primarily satisfy the demands of human activities by developing surface and groundwater resources through methods such as seawater desalination and resource allocation^7,8^. However, with the increasing demand for water resources, the volume of water provided by current acquisition methods is gradually becoming inadequate. Overdevelopment of surface and groundwater can also cause certain environmental damages. For instance, the extraction of groundwater can lead to land subsidence^9^. Regrettably, atmospheric water, as a widely distributed and renewable water resource, has not yet been effectively exploited^10^. Therefore, the development of atmospheric water resources may expand the channels for water acquisition and alleviate the problem of global water scarcity^11–14^.

**Fig. 1:**
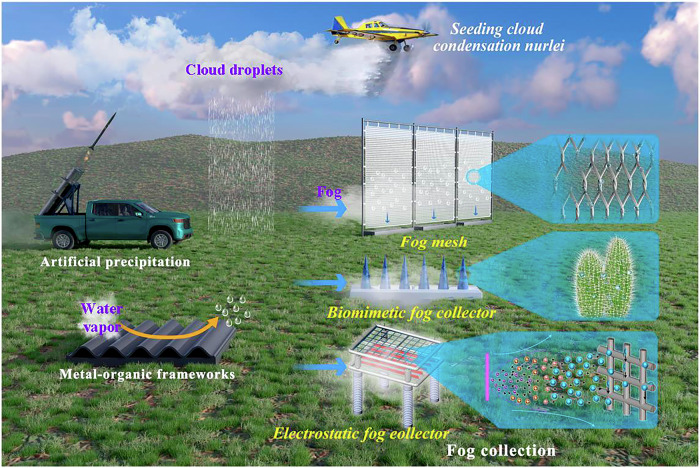
Methods for collecting atmospheric water^11,13,21,42,120–124^. For cloud droplets, they are usually formed into rain or snow by sowing condensation nuclei. For water vapor, humans collect it through moisture absorbing materials such as silica gel, calcium chloride, MOFs (Metal-organic frameworks) or condensation methods. For fog, humans generally set up fog collectors to make droplets collide and form water. Fog collectors are generally divided into fog mesh, biomimetic fog collectors, and electrostatic fog collectors according to their working principles. Fog mesh - use inertial collisions to intercept droplets. Biomimetic fog collectors - mimic biological structures to capture droplets (e.g., cactus thorns). Electrostatic fog collectors - utilize the charge and electric field generated by gas discharges to capture droplets.

Atmospheric water, characterized by its distribution, can generally be categorized into clouds, fog, and water vapor^15–17^. The characteristics and acquisition methods of different types of atmospheric water are summarized in Fig. 1 and Table 1. Compared to clouds and water vapor, fog has larger particle sizes (2 ~ 50 μm) and is closer to the ground (0 ~ 100 m)^18,19^. As shown in Fig. 1, fog water collection not only reduces reliance on groundwater and surface water but also has high adaptability for foggy and remote areas that lack other types of water resources (especially in mountainous and coastal regions): fog water collection requires relatively simple conditions, with fog collectors being easy to operate and maintain, and having low construction and operational costs, making them suitable for implementation in economically underdeveloped or remote areas^20–23^. As a renewable resource, the continuous collection of fog water does not significantly impact the ecosystem^12,24^. Moreover, the water quality of collected fog water is usually relatively pure, reducing the cost of subsequent treatment^22^. Against the backdrop of climate change, the increase in fog provides more opportunities for fog water collection, making it a potentially valuable method of water resource acquisition.

**Table 1:** Comparison of atmospheric water collection methods^45,55,108,109^

Methods	Atmospheric water	Particle size	Height above ground	Relative humidity	References
Artificial precipitation	Cloud	1 ~ 150 μm	600 ~ 2500 m	≥100%	^108^
Adsorption or condensation	Vapor	<3 nm	Whole atmosphere	20% ~ 80%	^109^
Fog collection	Fog	2 ~ 50 μm	0 ~ 100 m	>90%	^45,55^

Current methods for fog water collection are summarized in Table 2. Fog mesh collection pertains to the installation of mesh-type fog collectors (fog meshes or fog nets) in foggy environments, employing the inertial collision of fog droplets with the collector to intercept and form usable liquid water. The structure of the fog mesh is simple, easy to expand, and convenient for large-scale use. Many scholars have used fog nets to collect natural fog. For example, the fog collectors built in Chile, Morocco and other places all use fog mesh structures. However, this method collects fog water only by the inertial collision of fog droplets and fog collectors, and is limited by environmental factors such as temperature and dew point. Its collection efficiency is extremely low (1% ~ 2%). Although some scholars have made improvements to improve the collection efficiency (e.g., the efficiency of the harp structure is about 10%)^25–27^, the effect is not satisfactory.

**Table 2:** Summary of the characteristics of different types of fog collectors^46,47,77,110–117^

Type	Principle	Material	Ballpark (m^2^)	Collection location	Cost (USD)		Collection efficiency (%)	Collection rate (kg/m^2^/h)	Water cost (USD/L)	References
Fog mesh	Fog droplets inertia	Raschel	1	Chile	/		1% ~ 10%	0.06 ~ 0.5	0.03 ~ 0.08	^47,110–112^
		Raschel	40	Morocco	1000 ~ 1500					
		Spacer fabric	54	Canary Islands	13,000					
		Polyethylene	25	Chile	400					
Biomimetic fog collector	Imitation of biological microstructure	Spiderweb fiber	/	Indoor, ultrasonic fog source		/	15% ~ 30%	0.95 ~ 1.5	/	^113–117^
		Lotus leaf protrusion								
		Gunnera leaf								
Electrostatic fog collector (EFC)	Synergy between charge and electric field	Stainless steel	1	Indoor, ultrasonic fog source		4000 ~ 6000	50% ~ 90%	3.2 ~ 5.6	0.02 ~ 0.05	^46,77^
		Janus metal mesh	0.04							

To augment the quantity of fog collected, numerous scholars have enhanced the collection efficiency by emulating the microstructures of natural organisms (e.g., the spider web fibers, lotus leaf protrusions, and Gunnera leaf in Table 2), a method referred to as “biomimetic” collection. Although biomimetic collection can elevate the efficiency to a certain extent, it is excessively reliant on the microstructure of the collector’s surface; if the microstructure is impaired, the performance of the fog collector will notably deteriorate. Moreover, the fabrication process of biomimetic collection is also comparatively complex, entailing higher production costs. Currently, most of the research on bionic fog collectors is still in the experimental stage.

**Fig. 2:**
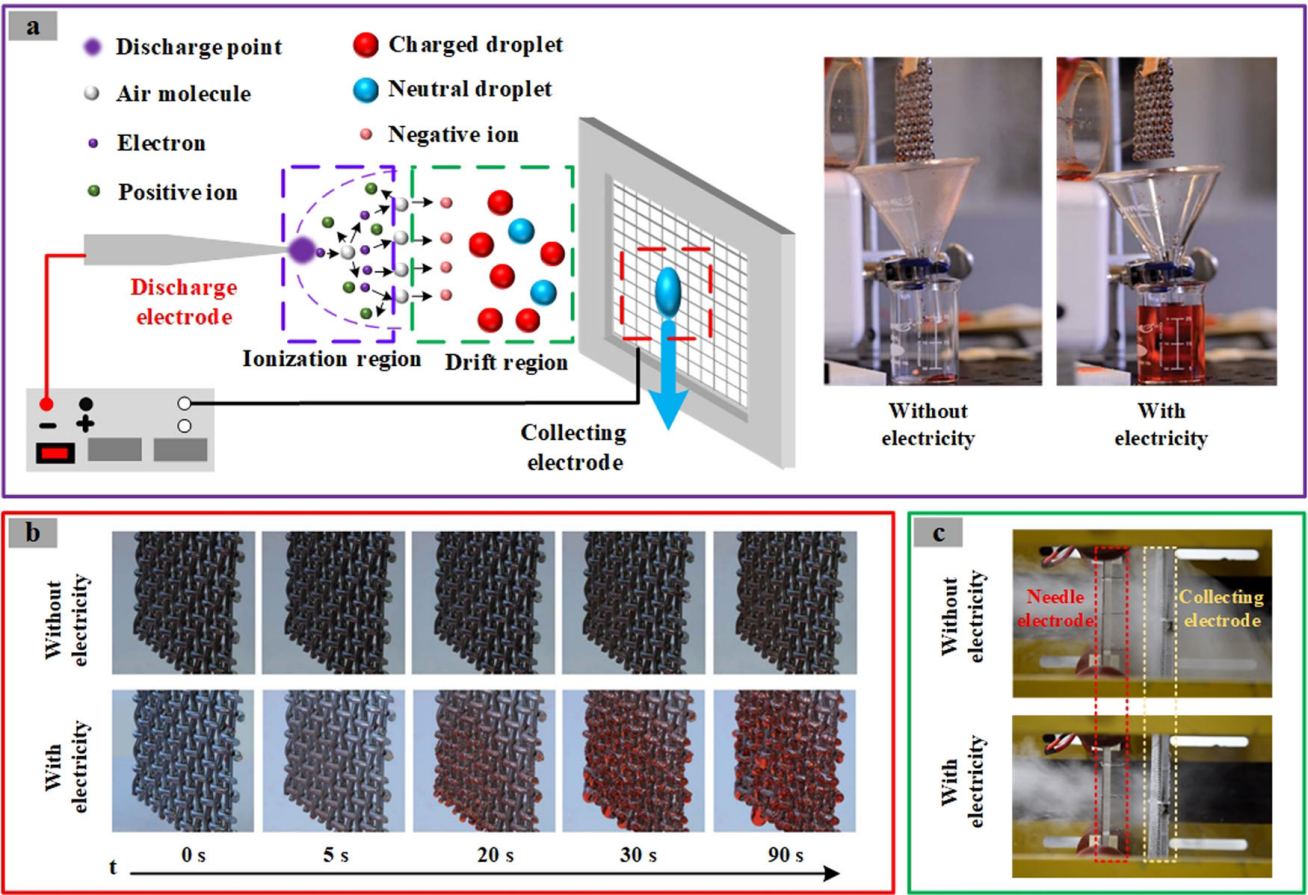
Comparison of fog water collection efficiency of EFC (Electrostatic fog collector) with and without electricity^30,77^. **a** Schematic diagram of the principle of the EFC and the photo to the right show the water collected over the same period of time for the EFC with and without electricity, respectively. (Reproduced with permission from ref. ^30^). **b** The condensation condition on the collecting electrode of EFC with or without electricity at different times (Reproduced with permission from ref. ^30^). **c** Images of fog droplets intercepted by EFC with and without electricity (Reproduced with permission from ref. ^77^).

In recent years, drawing inspiration from electrostatic dust collectors, some scholars have put forward a method to enhance fog water collection through the synergistic effect of charge and electric fields, a technique also referred to as “electrostatic fog water collection technology”^28–30^. The fundamental principle entails the generation of a strong electric field via gas discharge and the release of charged particles (including positive ions and negative ions, positive ions such as N_4_^+^, O_2_^+^, NO^+^, H_3_O^+^, CO_2_^+^, etc.; negative ions such as O_2_^−^, H_3_O^−^, NO^−^, CO_3_^−^, etc.) into the surrounding space to electrify fog droplets, which are subsequently captured by the fog collector under the influence of the electric field. After the charged droplets are captured by the collecting electrode, their charge is neutralized. The collection efficiency (50% ~ 90%), water yield (3.2 ~ 5.6 kg/m^2^/h) and cost of the electrostatic fog collector (EFC) are much higher than those of the other two types of fog collectors due to the strengthening effect of charge and electric field. As depicted in Fig. 2, compared with EFC without electricity, EFC with electricity has high collection efficiency and rapid effect, and is a sustainable fog water collection technology^30,31^.

To promote intellectual exchange across different research domains and to stimulate new research concepts, this article systematically examines the research trajectory of electrostatic fog collection technology and deliberates on the latest advancements in its enhancement research. Ultimately, we present a forward-looking research orientation for the future of electrostatic fog collection technology, with the aim of facilitating the transition from scientific research to practical application.

## Physical process and difficulties in efficiency enhancement

### Physical process

**Fig. 3:**
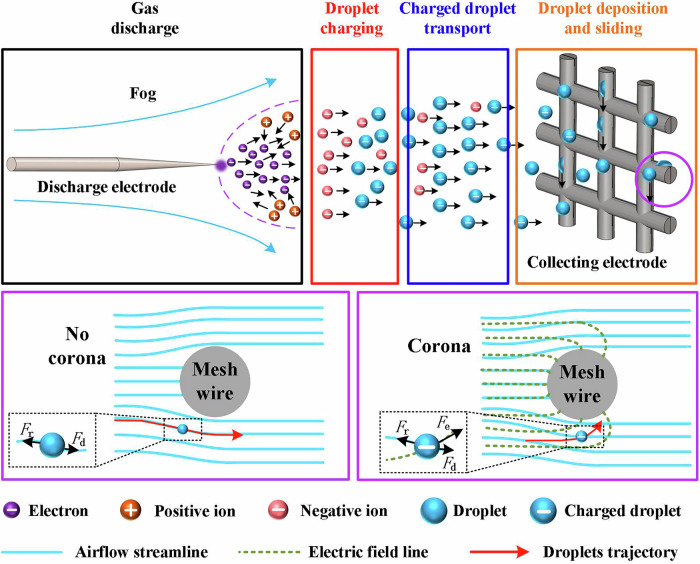
Schematic diagram of the basic principle of EFC (Electrostatic fog collector). *F*_r_-air resistance, *F*_d_-fluid drag, *F*_e_-electric field force.

Figure 3 is a schematic diagram of the basic structure and operating principle of EFC based on CD (corona discharge). The EFC consists of a power supply and electrodes. The power supply is a high-voltage source used to induce gas discharge; the electrodes include a discharge electrode and a collection electrode. The discharge electrode has a discharge tip with a small radius of curvature, while the collection electrode is a blunt electrode. The discharge electrode is connected to the power supply to ionize the background gas and generate space charges. It is also known as a “high-voltage electrode”. The collecting electrode is grounded and used to capture droplets and their charges. It is also known as the “ground electrode”.

The basic physical process of electrostatic fog collection is shown in Fig. 3, which includes four stages: Gas discharge, droplets charging, charge droplets transport, droplet deposition and sliding. Gas discharge: After the EFC is energized, the discharge electrode is violently enhanced by the extremely small curvature of the surrounding electric field, ionizing the air to produce charged particles, which move to the collection electrode under the action of the electric field^32–35^. Droplets charging: After the droplets enter the electric field, the charged particles generated by gas discharge combine with the droplets to form charged fog droplets. Charge droplets transport: The charged fog droplets, driven by both the electric field and the flow field, move towards the collection electrode. Droplets deposition and sliding: When charged droplets approach the collection electrode, the electric field force overcomes the drag and resistance of the airflow to capture them. These droplets aggregate into large water droplets on the electrode surface and flow into the water storage container for use under the action of gravity. The above process is discussed in detail below.

**Fig. 4:**
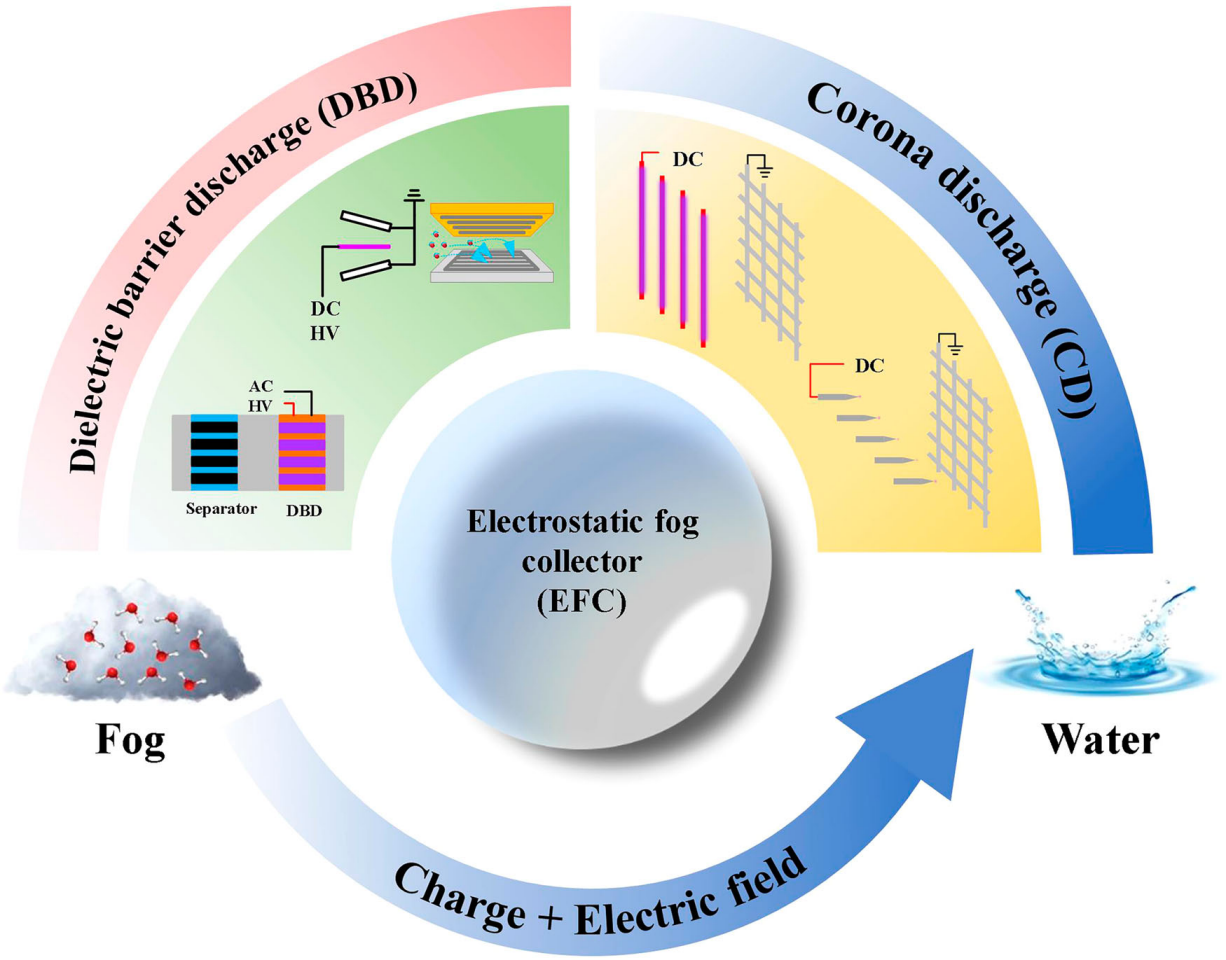
Common structures of electrostatic fog collectors (Reproduced with permission from refs. ^31,46,125^). The pattern discharge methods of EFCs are usually divided into DBD type and CD type. DBD-EFCs (Electrostatic fog collector based on dielectric barrier discharge) are generally composed of a charging device and a collector. The charging device charges the droplets, and the collector forms an electric field to capture the charged droplets. CD-EFCs (Electrostatic fog collectors based on corona discharge) are generally composed of discharge electrodes and collection electrodes. The discharge electrode is generally a sharp electrode, such as wire, needle electrode, etc; collect electrodes as blunt electrodes, such as plates, meshes, harps, etc.

The high collection efficiency of the EFCs can be attributed to two main reasons: Firstly, the charge increases the collision efficiency between fog droplets^36–41^. Secondly, the electric field accelerates the deposition of droplets, thereby expanding the interception area of the collection electrode^18,30,42^. In addition to corona discharge, EFC also generates charged particles through DBD (dielectric barrier discharge). Therefore, according to the type of discharge, EFC can be divided into two types: CD-EFC (Electrostatic fog collectors based on corona discharge) and DBD-EFC (Electrostatic fog collector based on dielectric barrier discharge), as shown in Fig. 4. Different from CD-EFC, the discharge electrode of DBD-EFC is composed of metal material and dielectric layer.

#### Discharge and droplet charging

Gas discharge is the working foundation of EFC, which provide high-density space charge and strong electric field for fog collection. Fog droplets, as particulate entities, typically exhibit two mechanisms of electrification: field-induced charging and diffusion charging^43,44^. Field-induced charging occurs when charged particles collide with fog droplets under the influence of an electric field, thereby imparting a charge to the droplets. The expression for the charge quantity in field-induced charging is presented as follows^18,19,30,45^:

1 $${q}_{{\rm{f}}}={\rm{\pi }}\left(\frac{3\varepsilon }{\varepsilon +2}\right){\varepsilon }_{0}{E}_{0}{d}_{{\rm{p}}}^{2}\left(\frac{1}{1+{t}_{0}/t}\right)$$

2 $${E}_{0}=\frac{U}{L}$$

3 $${t}_{0}=\frac{4{\varepsilon }_{0}}{{N}_{0}e{u}_{{\rm{i}}}}$$

Where, *q*_*f*_ represents the charge quantity due to field-induced charging; *ε* is the relative permittivity; *ε*_0_ is the vacuum permittivity; *E*_0_ is the charging electric field; *d*_*p*_ is the diameter of the fog droplet; *t* is the charging time; *t*_0_ is the time constant for field-induced charging; *U* is the voltage of the discharge electrode; *L* is the distance between the discharge electrode and the collection electrode; *N*_0_ is the ion concentration; *e* is the elementary charge; and *u*_*i*_ is the ion mobility. The process of field-induced charging primarily relies on the electric field to drive the collision of charged particles with fog droplets. When the accumulated charge on the fog droplets generates a strong electric field, the charged droplets will begin to repel particles of the same polarity. Consequently, the charge quantity in field-induced charging reaches a saturation value. It is generally considered that when the charging time *t* = 10*t*_0_, the charge quantity of the fog droplets reaches its saturation value. The saturation charge quantity of the fog droplets is given as follows^18,19^:

4 $${q}_{{\rm{s}}}=3{\rm{\pi }}{d}_{{\rm{p}}}^{2}{\varepsilon }_{0}{E}_{0}$$

Where, *q*_s_ denotes the saturation charge quantity of field-induced charging.

Diffusion charging is independent of the electric field intensity and refers to the process where fog droplets become charged by irregular Brownian collisions with charged particles. The expression for the charge quantity in diffusion charging is as follows^44^:

5 $${q}_{{\rm{k}}}={q}^{\ast }\,\mathrm{ln}\left(1+\frac{{t}_{1}}{{\tau }^{\ast }}\right)$$

**Fig. 5:**
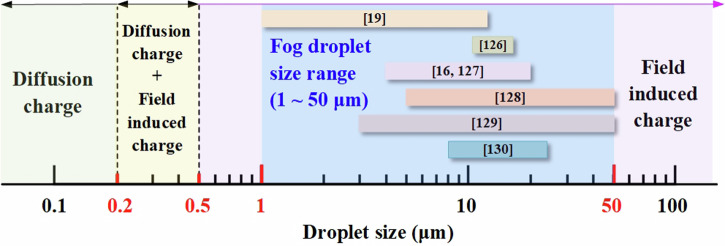
Relationship between the charging mechanism of fog droplets and the size of fog droplets^16,19,126–130^. Green area: the droplet particle size is less than 0.2 μm, and its main charging mechanism is diffusion charging. Yellow area: the droplet size is larger than 0.2 μm and smaller than 0.5 μm, and its main charging mechanism is diffusion charging and field charging. Purple region: the droplet particle size is larger than 0.5 μm, and its main charging mechanism is field charging. Blue region: the range of particle sizes of droplets produced by natural and commercial humidifiers.

Where, *q*_k_ represents the charge quantity due to diffusion charging; q^∗^ is the particle charge constant; t_1_ is the diffusion time; and τ^∗^ is the time constant for diffusion charging. The key factor governing the charging mechanism of fog droplets is the droplet diameter^44,46^. There are three typical cases of particle charging, as shown in Fig. 5. The specific details are as follows:

For droplets with a particle size greater than 0.5 μm, field-induced charging predominates. For droplets with a particle size less than 0.2 μm, diffusion charging is the dominant mechanism. For droplets with a particle size between 0.2 μm and 0.5 μm, both charging mechanisms are comparably effective^19^. Figure 5 also summarizes the diameter range of fog droplets previously studied. Whether from natural or commercial fog sources, the droplet diameter is greater than 0.5 μm, indicating that field induced charging is the main mechanism for droplet charging. According to Eq. (3), the charge quantity of the fog droplets is directly proportional to the electric field strength.

High fog droplet charge is one of the essential conditions for achieving efficient electrostatic fog collection. Although the amount of fog droplet charge increases with the supply voltage, the field induced charge motor system results in a saturation value for the amount of fog droplet charge. Indiscriminately increasing the discharge voltage also raises the requirements for equipment insulation, reducing operational safety. Moreover, the spatial charge density of the charged droplets varies significantly at different spatial positions, with the highest droplet charge density at the electrode axis and gradually decreasing along the radial direction.

Therefore, improving the discharge intensity of EFCs under low voltage conditions and accurately grasping the spatial charge distribution of the collectors is one of the necessary ways to enhance the efficiency of electrostatic fog collection technology.

#### Charged droplet transport

Charged fog droplets are transported towards the collection electrode under the combined action of the electric field and flow field, ultimately being captured by the collection electrode. The motion state of the charged fog droplets within the electric field determines their effective transport to the surface of the collection electrode. Therefore, analyzing the motion state of the charged fog droplets is a necessary pathway to enhance the efficiency of electrostatic fog collection. The motion state of the charged fog droplets is primarily determined by the electric field force *F*_*e*_ (the direction is that the discharge electrode points to the collecting electrode), the drag force *F*_*d*_ (the direction is consistent with the air flow direction), and the air resistance *F*_*r*_ (the direction is opposite to the direction of droplet movement), thus the motion equation of the charged fog droplets can be derived^18^:

6 $$m\frac{{\rm{d}}\overrightarrow{u}}{{\rm{d}}t}={\overrightarrow{F}}_{{\rm{e}}}+{\overrightarrow{F}}_{{\rm{d}}}+{\overrightarrow{F}}_{{\rm{r}}}$$

7 $${F}_{{\rm{e}}}=q{E}_{0}$$

8 $${F}_{{\rm{d}}}=3{\rm{\pi }}\mu {d}_{{\rm{p}}}{u}_{1}$$

9 $${F}_{{\rm{r}}}=3{\rm{\pi }}\mu {d}_{{\rm{p}}}u$$

Where, *q* is charges carried by the droplet; *μ* represents the dynamic viscosity of air; *u*_1_ is the airflow velocity; *u* is the velocity of the fog droplet; *m* is the mass of the fog droplet; *ρ* is the air density. By solving the differential equation, the expression for the velocity of the charged fog droplets can be obtained:

10 $$u=\left({u}_{0}-B/A\right){e}^{(-A\cdot t)}+B/A$$

11 $$A=\frac{3{\rm{\pi }}\mu {d}_{{\rm{p}}}}{m};B=\frac{(3{\rm{\pi }}\mu {d}_{{\rm{p}}}{u}_{0}+{q}_{{\rm{s}}}{E}_{0})}{m}$$

Where, *u*_0_ represents the initial velocity of the fog droplet. From Eq. (10)~(11), the motion state of charged droplets depends on electrical factors such as electric field and environmental factors such as wind speed and direction^46^. These factors directly determine whether the droplets can reach the collection electrode smoothly to achieve the capture of droplets. However, the non-uniform electric field formed by gas discharge may cause the droplets to encounter impediments in certain areas of the electrode gap, reducing collection efficiency. In addition, environmental factors such as temperature, humidity, and wind speed can also affect discharge performance, leading to a decrease in droplet charge.

To improve the efficiency of EFC by optimizing the motion state of charged droplets, it is not only necessary to accurately control the electric field distribution of the EFCs, but also to comprehensively consider environmental factors (flow field distribution, temperature, etc.) and the physical characteristics of the droplets. Through theoretical analysis, numerical simulation, and experimental research, a deeper understanding of the motion mechanism of the charged fog droplets can be achieved, and corresponding optimization strategies can be proposed.

#### Droplets collection

Collection efficiency *η* is a critical performance metric for fog collectors, determined by aerodynamic efficiency *η*_a_, deposition efficiency *η*_d_, and drainage efficiency *η*_s_.

12 $$\eta ={\eta }_{{\rm{a}}}{\eta }_{{\rm{d}}}{\eta }_{{\rm{s}}}$$

**Fig. 6:**
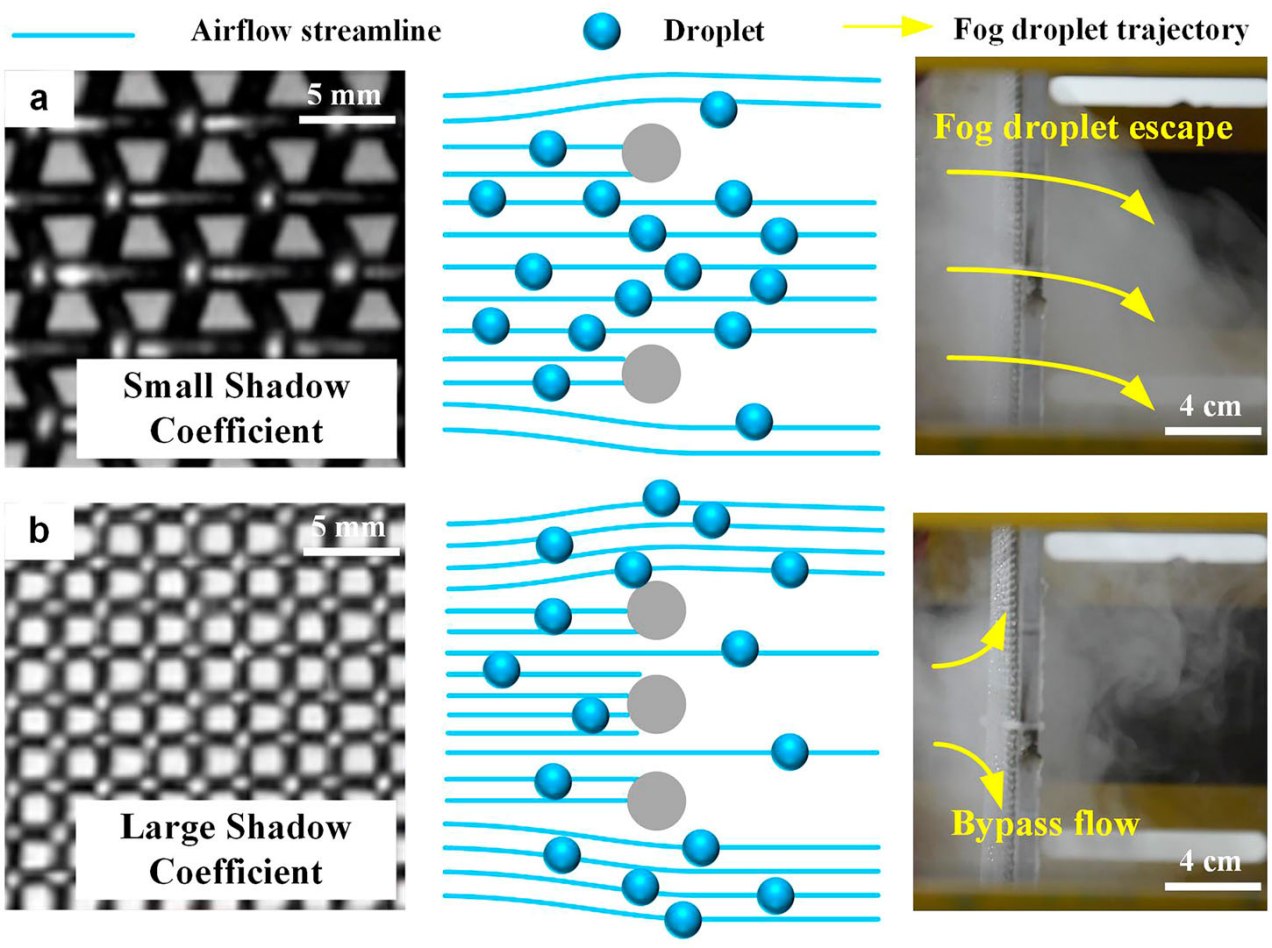
Schematic diagram of fog droplet collection using fog collectors with different shadow coefficients (Reproduced with permission from ref. ^21^). **a** Fog collector with small shadow coefficient, which collects water with a large number of fog droplets escaping. **b** Fog collector with large shadow coefficient, which collects water with the presence of fog bypassing the flow. A small shadow coefficient represents a large open area; a large shadow coefficient represents a small open area.

The aerodynamic efficiency of a fog collector refers to the ratio of the number of droplets moving towards the EFC to the total number of droplets^20^. It is related to the shadow coefficient of the collection electrode. The shadow coefficient is defined as the ratio of the fiber area to the total mesh area. As shown in Fig. 6, a shadow coefficient that is too small results in weak interception of droplets, leading to low collection efficiency; a large shadow coefficient will hinder the airflow and increase the turbulence rate of the fog. A large turbulence rate will cause bypass flow, resulting in a large number of droplets escaping.

According to previous research, when the shadow coefficient of the fog collector is 55%, the aerodynamic efficiency of the collector is maximized. However, this conclusion is only applicable to traditional fog mesh. For EFC, due to the intervention of electrical energy, the equivalent collection area of the fog collector has been expanded, as shown in Fig. 3. This leads to competition between the mesh wires of the collecting electrodes when capturing droplets, thereby reducing the collection efficiency^30^. Therefore, when optimizing the collection electrodes of electrostatic precipitators, the effect of electric field force needs to be considered.

The deposition efficiency of a fog collector, also known as the capture efficiency, refers to the ratio of the number of fog droplets captured by the fog collector to the number of droplets moving towards it. In an EFC, the deposition of fog droplets is the result of the electric field force overcoming drag and air resistance, as illustrated in Fig. 3. Therefore, the deposition efficiency is expressed as follows^30^:

13 $${\eta }_{{\rm{d}}}=c\frac{{\overrightarrow{F}}_{{\rm{e}}}}{{\overrightarrow{F}}_{r}+{\overrightarrow{F}}_{{\rm{d}}}}$$

Where, c represents an experimental constant. By substituting Eq. (7)~(11) into the aforementioned equation, the deposition efficiency of the electrostatic fog collector can be obtained^19^:

14 $${\eta }_{{\rm{d}}}=c\frac{{d}_{{\rm{p}}}{\varepsilon }_{0}{U}^{2}}{\mu L{R}_{{\rm{w}}}u(1+\frac{{d}_{{\rm{p}}}{\varepsilon }_{0}{U}^{2}}{u\mu {L}^{2}})}$$

Where, R_w_ denotes the radius of the wire of the collection electrode. From Eq. (14), although deposition efficiency can be improved by increasing the electrode voltage and optimizing the electrode size, the aforementioned measures only address the enhancement of charged fog droplet deposition. In actual collection processes, there are still some neutral or low-charge fog droplets. Therefore, how to improve the capture efficiency of neutral/low-charge fog droplets is also crucial for enhancing the efficiency of fog water collection.

#### Droplet slippage

The accumulation of fog droplets on the collection electrode to a certain extent leads to the formation of larger droplets. Unlike electrostatic dust collectors, large droplets can simply slide off into the collection container under the influence of gravity. The sliding-off process is generally characterized by drainage efficiency. The drainage efficiency of a fog collector refers to the ratio of the number of fog droplets collected in the container to the number of droplets deposited on the collection electrode, which is generally related to the surface morphology of the electrode. In other words, drainage efficiency is related to the hydrophobicity or hydrophilicity of the collection electrode surface^47^.

**Fig. 7:**
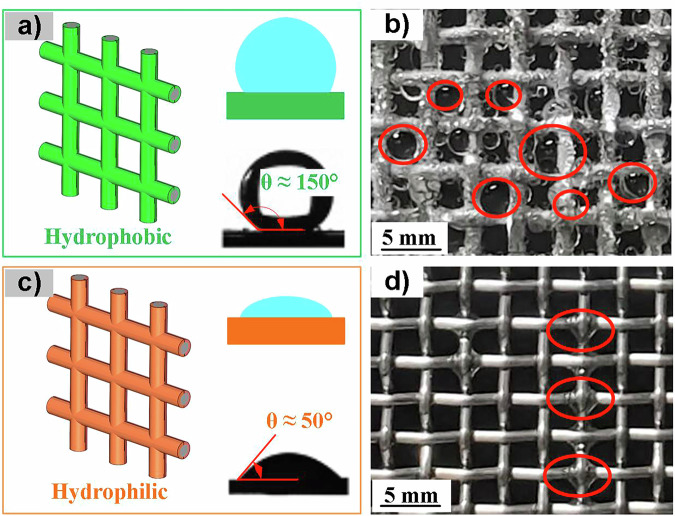
Residual liquid droplets in hydrophobic and hydrophilic fog collectors (Reproduced with permission from ref. ^77^). **a** Droplet contact angle of hydrophobic fog collector. **b** Image of residual droplets in hydrophobic fog collector. **c** Droplet contact angle of hydrophilic fog collector. **d** Image of residual droplets in hydrophilic fog collector. Red circles indicate residual droplets on the fog collector surface clogging the mesh in (**b**) and residual droplets failing to slide off in (**d**).

As shown in Fig. 7, if the hydrophobicity is too strong, although it can facilitate timely drainage, the large contact angle of the droplets on a hydrophobic surface can also cause changes in the shadow coefficient, reducing aerodynamic efficiency; if the hydrophilicity is too strong, it can cause many droplets to remain, preventing the collected fog water from being discharged in time, resulting in efficiency loss. Therefore, how to balance the hydrophobicity and hydrophilicity of the fog collector, to achieve high drainage efficiency while also minimizing the impact of accumulated droplets on the shadow coefficient of the collector, is of great significance for improving the efficiency of fog water collection.

### Efficiency enhancement methods

**Fig. 8:**
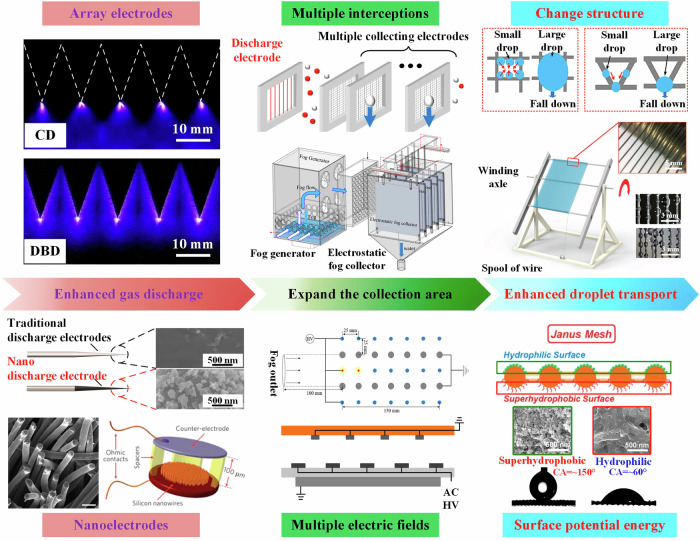
Efficiency enhancing methods of electrostatic fog water collection technology. Enhanced gas discharge can be achieved by increasing the density of discharge points and reducing the curvature radius of electrodes (Reproduced with permission from refs. ^84,118^). Expanding the collection area can be achieved by increasing the number of fog droplet interceptions (Reproduced with permission from refs. ^19,31,55,56,119^). Improving the transport speed of droplets can be achieved by changing the hydrophobicity of the collection electrode surface (Reproduced with permission from refs. ^26,60,70^).

As depicted in Fig. 8, the current methods to enhance the efficiency of EFCs are primarily threefold: enhanced discharge, expand the collection area, and enhanced droplet transport.

#### Enhanced discharge

The spatial electric field and charged particles generated by gas discharge directly affect the processes of fog droplet charging, migration, and collection. Enhancing gas discharge can increase the charge on fog droplets, amplify the electric field force, thereby improving the performance of the EFC. In electrostatic fog water collection, to electrify more fog droplets, employing a multi-electrode structure as the discharge electrodes of the EFC is an important means of increasing the collection efficiency, as shown in fig. S1 (Fig. S1 refers to Fig. S1 in the Supplementary Information)^48–53^.

Furthermore, Xiao et al. constructed a micro-nanostructure on the surface of a conventional discharge electrode (e.g., wire electrode), as shown in Fig.  S2a^45^. This method can organically unify small discharge tips and high-density discharge points. By strengthening gas discharge, the efficiency of fog collection is improved, as shown in Fig.  S2b^45^. As depicted in Fig. S3c, the corona onset voltage of the discharge electrode with micro- and nanostructures was reduced by approximately 46%, and the maximum current enhancement rate was about 40%. The increase in discharge intensity also increases the charge on fog droplets at the same voltage, thereby improving the efficiency of fog water collection^45,54^.

However, the fog collection efficiency does not increase indefinitely with the discharge intensity. When the electric field force on the charged fog droplets is balanced with the drag force and air resistance, the fog collection efficiency will reach a plateau value. The minimum voltage corresponding to the saturated fog collection efficiency is referred to as the “saturation collection voltage”^30,46^. Therefore, when operating the fog collector, it is important to control the operating voltage near the saturation collection voltage to achieve maximum collection efficiency while ensuring that the voltage of the fog collector is not excessively high, which could reduce the safety of the equipment.

In summary, enhancing gas discharge can effectively increase the charge on fog droplets and the electric field force, thereby achieving an increase in electrostatic fog collection efficiency. However, due to the force balance of charged droplets in the electric field, simply strengthening discharge cannot continuously improve the fog collection efficiency. In the practical application of the EFCs, droplets cannot be fully charged, which is one of the important reasons why the collection efficiency cannot be continuously improved with the discharge intensity. To further improve the collection efficiency, it is necessary to enhance the ability of the fog collector to capture neutral/low charge droplets.

#### Expand collection area

In addition to enhancing the efficiency of EFC by enhanced gas discharge, some scholars have also proposed to improve the efficiency of fog water collection by expanding the collection area of EFC, as shown in Fig. S3^18,54–56^. Yan et al. proposed a novel EFC consisting of a discharge electrode, the repulsion electrodes, and the collection electrodes, which theoretically achieves complete collection of fog water, as shown in fig. S3a~d^55^. The EFC utilizes the electric field formed between the repulsion electrodes and the collection electrodes to intercept the fog droplets multiple times. Yuan et al. demonstrated the high efficiency and feasibility of the novel EFC by achieving a collection efficiency of 90% by experimental validation^56^. Zhang et al. replaced the collection electrode of the EFC with a harp-like structure, as shown in Fig. S3e, f^54^. The harp style EFC uses a multi-layer staggered arrangement to collect fog based on the characteristics of fluid distribution, with a collection efficiency of about 80%^54^. Li et al. proposed an array type EFC, which achieves multiple interception of droplets by increasing the number of collection electrodes. The research results indicate that the 5-layer collection electrode can increase the collection efficiency of EFC from 55% to 82%^19^. Thus, the advantage of the multi collection electrodes is that the EFC can intercept droplets multiple times, expanding the collection area of the EFC. However, this method comes at the cost of sacrificing space in exchange for high fog water collection efficiency, which is not conducive to the miniaturization and integration of the device.

In addition to increasing the number of collection electrodes to expand the collection area, it can also be achieved by changing the motion state of charged droplets. Zhang et al. proposed a fog water collection method utilizing the convection of ion wind to enhance the collection efficiency, with the basic principle illustrated in fig. S3g^18,57,58^. As shown in fig. S3h, a power supply provides electricity to the charging electrode and the interception electrode, resulting in corona discharge. Fog droplets enter the electric field from charged electrodes and are charged by charged particles. Charged droplets reach the collection electrode under the drive of an electric field, achieving their first collection. Subsequently, fog droplets that were not collected for the first time entered the interception region. The interceptor electrode generates an ionic wind in the opposite direction of the original fog. Under the action of the reverse ion wind and electric field generated by the interception electrode, the fog droplets are once again captured by the collection electrode. Compared to traditional electrode structures, the advantage of this configuration is that the reverse ion wind can intercept fog droplets that were not collected in the first instance, increasing the effective collision area between the fog droplets and the collection electrodes. The fog water collection rate of this method is twice that of traditional EFCs. However, the additional introduction of interception electrodes to excite the ion wind increases the operating power of the fog collector.

In summary, although expanding the collection area can effectively improve fog collection efficiency, it still presents issues such as spatial waste and high-power consumption. The additional introduction of high-voltage power supplies not only increases the cost of the fog collector but also raises the requirements for its insulation properties. It is evident that enhancing the collection efficiency of single-layer electrodes and reducing operating voltage are crucial for the integration of EFCs.

#### Enhanced droplet transport

In electrostatic fog water collection, the transport speed of droplets on the collection electrode is directly related to the drainage efficiency of the fog collector and the aerodynamic efficiency during the collection process. Additionally, if the accumulated droplets on the collection electrode are not transported in a timely manner during the collection process, it will change the shadow coefficient of the EFC, which will reduce the aerodynamic efficiency. Therefore, some scholars have also proposed enhancing the collection efficiency by improving the hydrophobicity or hydrophilicity of the fog collectors^59^.

As shown in fig. S4a~c, Rajaram et al. enhanced the fog collection efficiency by approximately 50% by treating the inverted trapezoidal fog collection mesh with a superhydrophobic coating^60^. This inverted trapezoidal mesh structure, with its sloping edges and combined with a superhydrophobic coating, can rapidly transport droplets on the collection mesh, preventing droplet residue and blockage of the mesh pores. Shi et al. employed a harp-like fog collector to structurally prevent droplet residue, as depicted in fig. S4d~g^25,26^. The harp structure abandons the orthogonal structural characteristics of mesh-type fog collectors, reducing the droplet sliding volume to 0.05 μL, which avoids droplet residue on the collector and enhances the collection efficiency by about 10%. However, due to the lack of mechanical reinforcement from horizontal wire structures in the harp design, it may cause string vibration (such as in a vibrating string grid) under high wind speeds, leading to changes in the already optimized shadow coefficient.

To mitigate the negative impacts caused by structural changes, many scholars have proposed accelerating droplet transport by altering the surface energy of the fog collector, thereby alleviating the loss in collection efficiency due to changes in the shadow coefficient caused by residual droplets during the collection process. As illustrated in fig. S5a~e, Daniel et al. enhanced the surface energy of polycarbonate fibers (PC) by controlling the polarity of the voltage and humidity, thereby improving their fog collection capability^61^. The research results indicated that a high surface energy can increase the transport speed of droplets, increasing the collection efficiency by 46% to 145%. Enhancing the surface energy of the fog collector can also be achieved through micro-structures. As shown in fig. S5f~i, Feng et al. utilized 3D printing technology to construct asymmetric pillar tip structures, achieving directional transport of droplets^62^. Furthermore, Wang et al. leveraged the charge gradient and electric field gradient formed by corona discharge to facilitate the downward transport of droplets^63^. Leo et al. developed a T-shaped superhydrophobic surface with a droplet contact angle as high as 160°^64^. Li et al. employed a superhydrophobic stainless steel mesh for fog collection, where the contact angle of droplets on the collector surface reached up to 155°, reducing the adhesion of droplets to the fog collector and increasing the collection rate by 93%^65^. The aforementioned studies indicate that accelerating the transport of droplets on the surface of the fog collector can effectively improve the efficiency of fog water collection. However, during the collection of fog droplets, the excessively large contact angle of droplets on hydrophobic surfaces can still alter the shadow coefficient of the collector, leading to a loss in aerodynamic efficiency^25,26,60^.

To balance the strong droplet transport and the smaller contact angle of droplets, Janus structures with asymmetric wettability—where the windward side is hydrophobic (HB) and the leeward side is hydrophilic (HL)—have been widely applied to fog collectors^66–69^, as shown in fig. S6a~e. The Janus structure takes advantage of the difference in surface tension between hydrophobic and hydrophilic surfaces to drive droplets from the hydrophobic side to the hydrophilic side^70^. Additionally, the hydrophilic side of the Janus structure can also enhance the capture capability for fog droplets. Furthermore, the hydrophilic surface of the Janus structure can enhance the capture capability for fog droplets. As shown in fig. S6f, Wang et al. adjusted the parameters of femtosecond laser pulses to transform the wettability of polyimide (PI) films into superhydrophobic or superhydrophilic states, achieving droplet actuation^71^. Hou et al. designed an anisotropic Janus membrane with opposite wettability and a unique interpenetrating microstructure at the interface, demonstrating the “diode-like” performance of unidirectional liquid penetration^72^. Chen et al. constructed a Janus grid using superhydrophobic / superhydrophilic materials that enabled unidirectional droplet transport^70^. Zhang et al. developed an asymmetric wettability surface combined with self-driven triboelectric adsorption; the surface generated charges through friction to adsorb fog droplets and used asymmetric wettability to drive the droplets, ultimately achieving a water collection efficiency of 93.18 kg/(m^2^ h)^73^. As depicted in fig. S6g, Li et al. successfully designed a quadruple biomimetic Janus composite material with asymmetric microtopography and anisotropic wettability through chemical etching and electrospinning methods^74^. This material achieved a fog collection efficiency of 48.34 kg/(m^2^ h) through effective fog capture and unidirectional droplet transport^74^. Based on the aforementioned research approach, Janus composite structures can be applied to the collection electrodes of EFCs. However, since the operation of EFCs generates a significant amount of space charge, the materials used to construct the Janus structure must possess good electrical conductivity to prevent charge accumulation that could lead to electrode breakdown^61,68,75,76^.

In summary, to enhance the collection efficiency of EFCs from the perspective of droplet transport, it is necessary to consider both the transport rate of the deposited droplets on the electrode and the contact angle comprehensively. This ensures that the collection electrodes not only have a high drainage efficiency but also minimize the impact of droplets on the electrode’s shadow coefficient during the collection process.

#### Composite efficiency enhancement

Based on the aforementioned research, it is evident that both intensifying gas discharge and increasing the droplet transport speed on the fog collector can improve collection efficiency to some extent. Consequently, Li et al. proposed an electrostatic fog collection method that couples plasma with micro-nano materials, as shown in fig. S7^77^. This method involves modifying the discharge electrode and collection electrode of EFC separately by nanotechnology. For discharge electrodes, they use metal nanoparticles to construct high-density nanoscale discharge points on the electrode surface, enhancing its discharge performance. For the collection electrode, the Janus composite structure (hydrophobic/hydrophilic) is constructed on the surface. This composite structure can generate a surface tension difference at the interface between HB (hydrophobic) and HL (hydrophilic), driving the droplets on the collection electrode surface from HB to HL in a directed manner. By adjusting the accumulation of droplets on both sides of the electrode, it effectively prevents issues of droplet residue and mesh clogging. This approach has reduced the saturation collection voltage and power of the EFC by about 30%, and increased the collection efficiency of the single-layer collection electrode to 93%^77^. The specific reasons for the excellent results achieved by this composite enhancement method are as follows^77^:

1. The metal nanoparticles on the discharge electrode have the high density of nanoscale discharge tips, which can effectively lower the voltage threshold for corona discharge, allowing the droplets to reach saturation of charge at a lower voltage. The electric field is enhanced by three orders of magnitude, and the ion concentration is increased by one order of magnitude.
2. The Janus composite structure on the collection electrode significantly enhances the transport capability of droplets on the collection electrode during the fog water collection process, effectively alleviating issues that lead to reduced collection efficiency, such as droplet residue and clogging of the collection electrode mesh pores.
3. The hydrophilic layer on the collection electrode significantly enhances the capture capability of the electrode for low-charge droplets and neutral droplets.

In summary, the use of nanoelectrodes not only significantly improves the efficiency of EFCs but also reduces their voltage and power consumption, providing a promising direction for the development of new types of EFCs in the future. However, the high concentration of reactive particles produced by gas discharge may have adverse effects on nanoelectrodes, such as plasma active particles accelerate the oxidation of nanomaterials. Whether nanoelectrodes can be continuously applied in a high-humidity discharge environment requires further research. Moreover, the mechanism of nanoelectrode discharge in a sustained high-humidity environment, as well as the exploration of economical and reliable methods for the preparation of nanoelectrodes, will become hot topics in this field of research.

The characteristics of different enhancement methods are summarized in Table 3. These methods improve the fog water collection efficiency by increasing the charge of droplets, expanding the collection area, and improving droplet transmission. In the future, we may be able to combine these methods to develop a new EFC to continuously provide water for humans.

**Table 3:** Summary of different methods to enhance EFC efficiency^18,19,21,26,55,56,60,77,84,118,119^

Methods	Type	Advantages	Limitation	References
Enhanced gas discharge	Array electrodes	Expanding the electric field area	Mutual interference	^118^
	Nanoelectrodes	Reduce operating voltage and power	Short lifespan	^77,84^
Expand the collection area	Multiple interceptions	High collection efficiency	Large area	^19,56,119^
	Multiple electric fields	High collection efficiency	Additional power supply	^18,55^
Enhanced droplet transport	Change structure	Rapid droplet transport	Weak mechanical strength	^21,26,60^
	Surface potential energy	Avoid residual droplets	Micro-nano structure destruction	^70,74^

### Future development trends

**Fig. 9:**
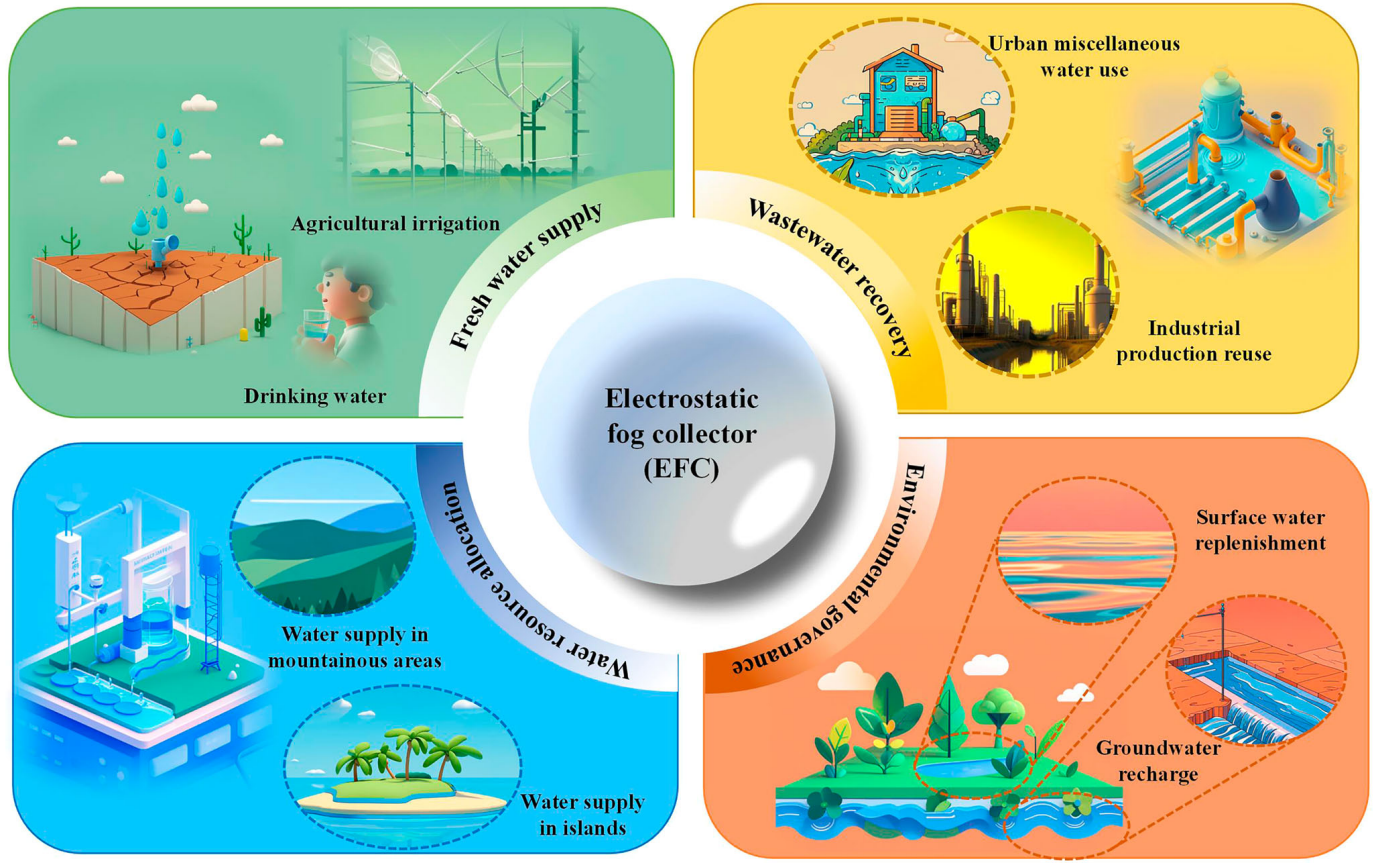
Application field of electrostatic fog water collection technology. Building upon the research findings of predecessors, we believe that this technology will evolve towards a future characterized by low voltage, low power consumption, high efficiency, strong durability, and high integration. At present, EFCs still face challenges such as high operating voltage, high power consumption, electromagnetic interference between fog collecting units, weak electrode durability in high humidity environments, and further improvement in insulation. We have proposed different solutions to address the above challenges to achieve large-scale application of electrostatic fog collection technology.

To keep pace with the global trend of low-carbon development and to accelerate the implementation of UNSDG, electrostatic fog water collection technology is poised to become an important means for humans to obtain water resources. This novel water resource acquisition technology will have great application prospects in areas such as freshwater supply, secondary use of water resources, water resource allocation, and ecological protection in the future, as shown in Fig. 9.

#### Low voltage and low power consumption

As is well known, gas discharge is the foundation of EFC operation. Therefore, intensifying the gas discharge is conducive to reducing its operating voltage and power consumption. Currently, there are two primary methods for intensifying gas discharge: increasing the number of discharge points and enhancing electrode curvature^78,79^.

With the rapid development of micro-nano technology, micro-nano electrodes can organically unify these two methods, breaking through the technical barriers of traditional electrodes^80–82^. As shown in Fig. S8, some scholars have used nanotechnology to prepare micro-nano electrodes suitable for gas discharge to improve discharge performance. Micro-nano electrodes have a high density of micro-nanoscale discharge tips that are prone to cause electric field distortion and high localization^83^, allowing for gas discharge at lower voltages. They hold broad application prospects in the development of low-energy consumption, high-efficiency, and integrated precision discharge devices^84–93^.

Currently, nanoelectrodes used for gas discharge are mostly fabricated using dispersion methods of micro-nano particles or vapor-phase growth methods, which often result in lower mechanical strength and susceptibility to structural damage during use^87^. Future efforts could explore the use of laser etching, 3D printing, and other advanced manufacturing techniques to produce micro-nano electrodes with enhanced durability and precision^62,94^. In addition, it is essential to conduct research on the discharge mechanisms of high-density micro-nano electrodes to understand their spatiotemporal discharge characteristics and the relationship between discharge intensity and the geometric features of the micro-nano electrodes (such as tip size, distribution characteristics, etc.), guiding the development of high-performance micro-nano electrodes.

#### Gas discharge mechanism in high humidity environment

Due to the unique operating environment of the EFCs (high humidity conditions), it is necessary to conduct research on the mechanisms of gas discharge under high humidity conditions^95,96^. Exploring the products of gas discharge in high humidity environments, the influence of active particles on electrodes, the evolution of discharge characteristics, and the influence of micrometer sized droplets on discharge characteristics by numerical simulation, cloud chamber discharge experiments, and other methods. These research areas can help deepen the understanding of the principles of electrostatic fog water collection technology and provide a scientific basis for the design of the EFCs.

#### Wide fog spectrum and efficient fog collection scheme

Currently, fog collectors operate in environments with high concentrations of fog, targeting fog droplets larger than 1 μm. However, in addition to droplets with a particle size greater than 1 μm, the air also contains sub-micron and nanoscale droplets. Therefore, expanding the particle size range (fog spectrum) of EFCs for collecting fog droplets plays an important role in further improving fog collection efficiency. Here, we propose two technical approaches, as shown in Fig. S9^38,43,97–99^.

First, by integrating the collection electrodes of the EFC with hygroscopic materials, such as metal-organic frameworks (MOFs), the combination of electrical charge and the hydrophilic nature of the hygroscopic material can enhance the collection of smaller diameter fog droplets^11,13^.

Second, an aerosol generator can be placed in front of the EFC to cause small fog droplets to coalesce and grow into larger droplets, which are then transported to the EFC for collection. In addition, researchers also need to explore the mechanisms by which charged particles promote the coalescence and growth of nanometer-sized droplets^63,100^, providing theoretical guidance for efficient broad-spectrum water vapor collection schemes under efficient conditions .

#### Research and development of new electrodes

Developing new high-performance electrode materials is crucial for enhancing the response speed, discharge performance, and service life of the EFCs. For the discharge electrode, the focus should be on the material’s secondary electron emission coefficient, chemical properties, mechanical strength, ozone concentration, and feasibility of integration with nanomaterials^101^. For the collection electrode, the emphasis should be on the material’s hydrophilicity/hydrophobicity, mechanical strength, and plasticity^36,67,68^.

#### Research and development of self-powered systems

Electric energy drive is a prerequisite for the operation of the EFCs, and the installation and debugging of energy supply systems pose challenges to the large-scale application of electrostatic fog collection technology. If the wind and light conditions of the application environment of the fog collectors can be utilized, it may promote the promotion of electrostatic fog collection technology. For example, using wind energy from foggy areas such as mountainous areas and islands to drive wind turbines to power electrostatic precipitators, as shown in Fig. S10^102^. In addition, the EFCs can also be powered by a solar cell. Integrating environmental detection equipment, energy storage equipment with power generation equipment to achieve dynamic control of electrostatic fog collection systems. When there is no fog, the self-powered system utilizes wind energy, solar energy, and ocean energy to generate electricity and store it. When there is fog, the self-powered system supplies energy to the EFCs^102^.

#### Integrated products

Developing integrated EFCs is an essential path for promoting the electrostatic fog water collection technology. Currently, some EFCs utilize an array of electrodes with multiple discharge points, which effectively expands the charging area of the fog collectors^50^. However, the mutual inhibitory effect between the discharge points in the array can weaken the overall discharge performance of the array electrodes, as shown in Fig. S11^103–107^. To achieve large-scale integration of the EFCs, in addition to the development of new electrode materials, broad-spectrum high-efficiency collection schemes, and self-powered systems, it is also necessary to address the insulation and electromagnetic compatibility issues of the EFCs^103,104^.

## Conclusion

The development and application of electrostatic fog collection technology have furnished new ideas and tools for the sustainable management of global water resources. It not only underpins water security in arid regions but also facilitates environmental protection, economic development, and social well-being. As the technology continues to progress and innovate, electrostatic fog water collection is anticipated to play an increasingly crucial role in future global water resource strategies. This article conducts a comprehensive review of the pertinent content of electrostatic fog collection technology, offering readers a thorough understanding of this domain. We have carried out an in-depth analysis of the physical process and efficiency enhancement methods of this technology, providing a theoretical foundation for further research and application. Additionally, we have provided an outlook on the future development trends of this technology and proposed targeted solutions to the existing issues in the current stage, which can offer guidance for research in related fields. The specific conclusions are as follows:

1. The fundamental physical process of electrostatic fog water collection technology encompasses four stages: gas discharge, fog droplet charging, charged droplet transportation, and droplet deposition and sliding. The device that implements this technology is termed an electrostatic fog collector (EFC). The primary factors influencing the efficiency of EFCs are the charge quantity of the fog droplets and the electric field in which the charged droplets are located, both of which are associated with the environment, electrode structure, and electrical parameters.
2. The collection efficiency of EFCs is governed by aerodynamic efficiency, deposition efficiency, and drainage efficiency. The shadow coefficient of the fog collector dictates the aerodynamic efficiency; however, it is imperative to mitigate the impact of residual droplets on the shadow coefficient during the collection process. The deposition efficiency is associated with the electric field force of charged droplets. It is of crucial importance to enhance the deposition efficiency by increasing the droplet charge and augmenting the capture of neutral/low charge droplets by the fog collector. The drainage efficiency is related to the hydrophilicity and hydrophobicity of the fog collector. Balancing the hydrophilicity and hydrophobicity of the fog collector allows it to possess high drainage efficiency while also minimizing the influence of liquid droplets on the shadow coefficient of the fog collector.
3. Currently, the methods for enhancing the efficiency of EFCs primarily encompass three approaches: intensifying gas discharge, expanding the collection area, and reinforcing droplet transport. Enhanced gas discharge is directed at increasing the charge rate of droplets and augmenting the electric field force of charged droplets. Expanding the collection area can attain multiple interception of droplets. Strengthening droplet transport can avert the loss of collection efficiency caused by droplet residue on the EFCs.
4. The future development of electrostatic fog water collection technology is poised to exhibit a trend towards low voltage, low power consumption, high efficiency, strong durability, and high integration. To attain the aforementioned objectives, it is believed that research should be carried out in six main areas: low-voltage low-power technical solutions, the mechanisms of gas discharge in high-humidity environments, broad-spectrum high-efficiency fog collection schemes, high-performance electrode materials, self-powered systems, and the development of integrated cost-effective products. Furthermore, general research ideas have been proposed for these directions, with the aim of facilitating the transition of electrostatic fog water collection technology from scientific research to practical application.

## Supplementary information

Supplementary Information

## Data Availability

The authors declare that all data supporting the findings of this study are available within the paper and its Supplementary information.
