# Supplementary material for: Sustainable solutions for water scarcity: a review of electrostatic fog harvesting technology
Source: Commun Eng. 2025 Feb 26;4:34. doi: 10.1038/s44172-025-00381-x (PMC11865594; doi:10.1038/s44172-025-00381-x)
Supplement: Supplementary file 1 — Supplementary Information [file 44172_2025_381_MOESM1_ESM.pdf]

Supplementary Information for

Sustainable Solutions for Water Scarcity: A Review of Electrostatic Fog Harvesting Technology

Dingchen Li, Chuan Li, Menghan Xiao, Ming Zhang, Jiawei Li, Zhiwen Yang, Qixiong Fu, Kexun Yu, Yong Yang, Yuan Pan, Yaping Du, Xiangen Zhao

*Corresponding author: Chuan Li, lichuan@hust.edu.cn

This PDF file includes:

Fig. S1| Different multi electrode structures.

Fig. S2| Schematic diagram of micro/nano structure enhanced electrostatic fog collection.

Fig. S3| Method of expanding the collection area to achieve EFC efficiency enhancement.

Fig. S4| Collection principle and efficiency of fog collectors with different structures.

Fig. S5| Different directional transport droplet materials and water collection performance.

Fig. S6| Principle and fog collection performance of Janus fog collector.

Fig. S7| Method for enhancing the collection efficiency of EFC by composite nanomaterials.

Fig. S8| Comparison of discharge currents between different micro/nano electrodes and traditional electrodes.

Fig. S9| Expanding the particle size range of EFC applied droplets to improve fog collection efficiency.

Fig. S10| Development diagram of EFC's self-powered system.

Fig. S11| Interaction diagram between discharge points during gas discharge with array electrodes.

References for Supplementary information

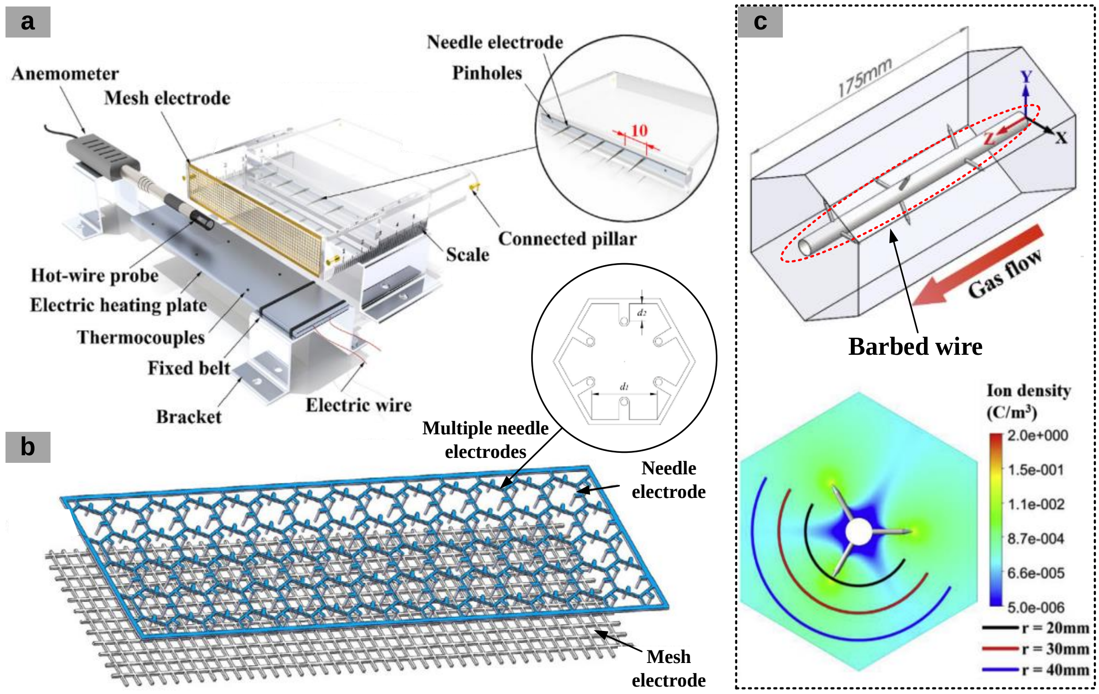

Fig. S1| Different multi electrode structures [1-3]. **a,** array needle - mesh electrode (Reproduced with permission from ref. 2). **b,** honeycomb needle - mesh electrode (Reproduced with permission from ref. 1).

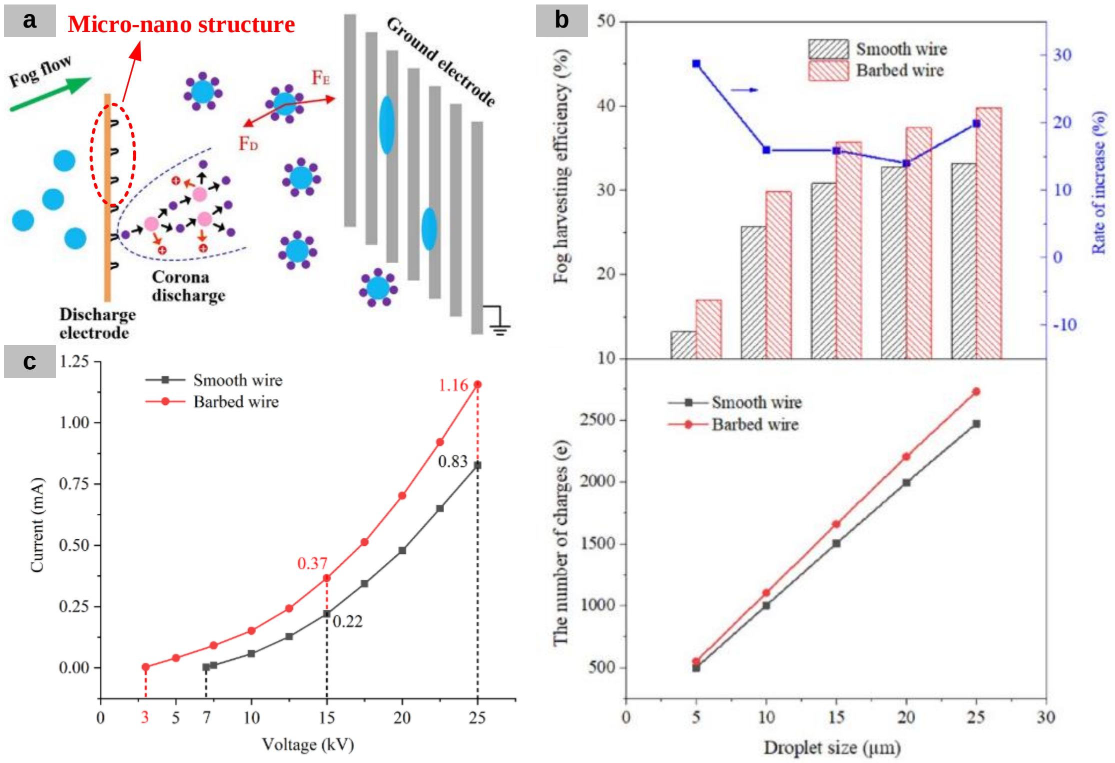

Fig. S2| Schematic diagram of micro/nano structure enhanced electrostatic fog collection [4,5]. **a,** the

principle of micro/nano structure enhanced fog water collection (Reproduced with permission from ref. 4). **b,** the collection efficiency and droplet charge under different operating conditions (Reproduced with permission from ref. 4). **c,** the discharge characteristics of smooth wire and barbed wire (Reproduced with permission from ref. 4).

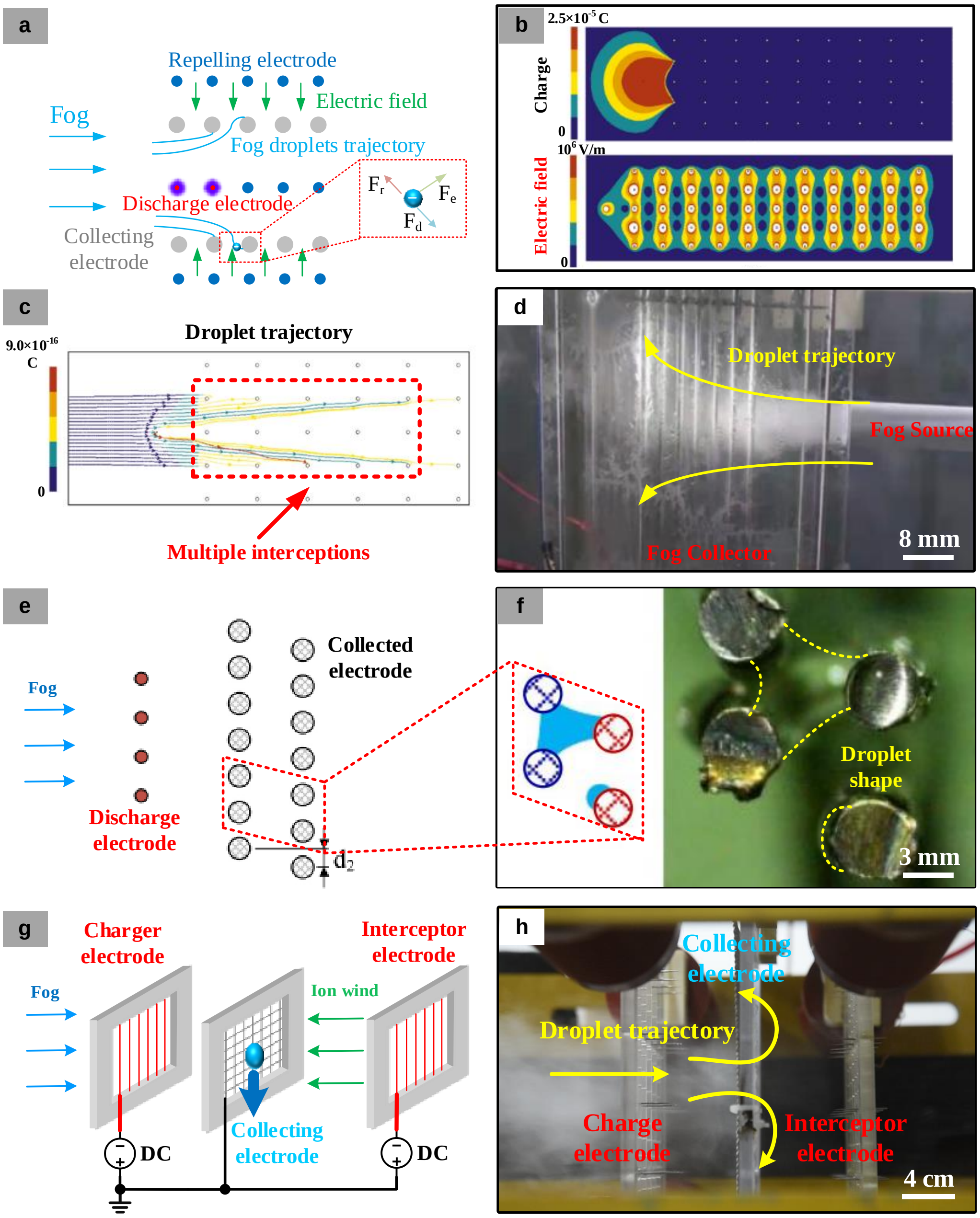

Fig. S3| Method of expanding the collection area to achieve EFC efficiency enhancement. a, discharge electrode -repelling electrode - collection electrode (Reproduced with permission from ref. 6). **b,** the electric field and charge density distribution of multiple repelling electrodes (Reproduced with

permission from ref. 6). **c,** multiple repulsive electrode droplet trajectories (Reproduced with permission from ref. 6,7). **d,** image of collecting fog with multiple repulsive electrodes (Reproduced with permission from ref. 7). **e,** harp style EFC (Reproduced with permission from ref. 4). **f,** image of droplet condensation on the harp style collection electrode (Reproduced with permission from ref. 4). **g,** ionic wind convection EFC (Interceptor electrode for generating reverse ionic wind)**. h,** image of ion wind convection EFC collecting fog water (Reproduced with permission from ref. [4,6-8]).

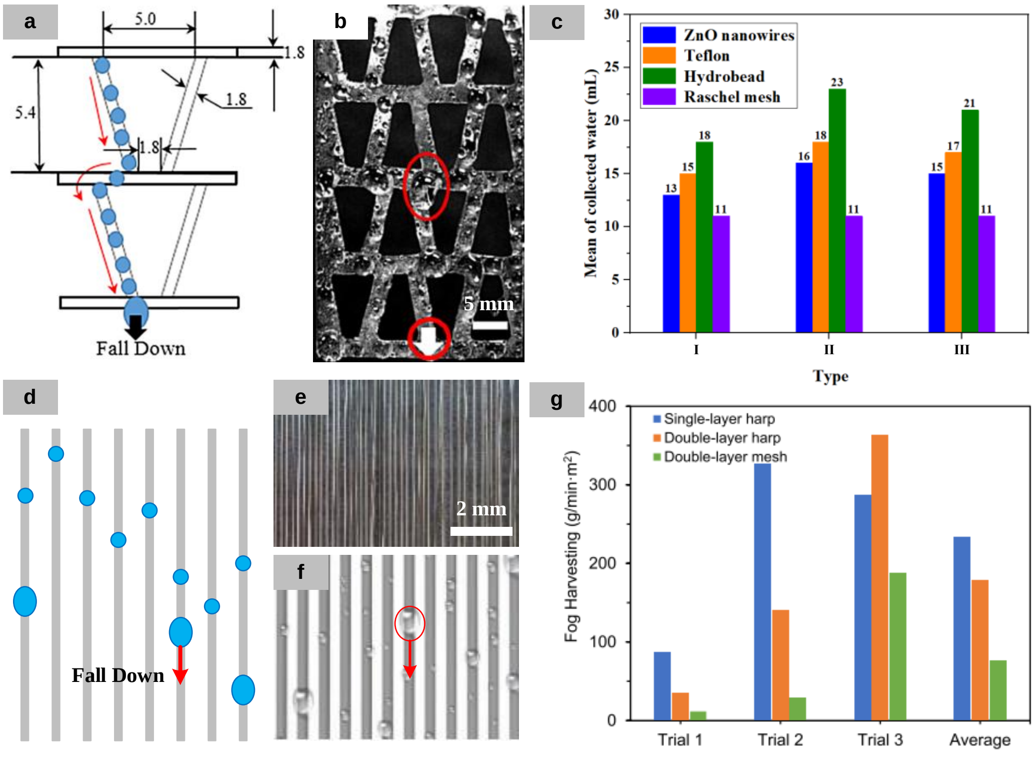

Fig. S4| Collection principle and efficiency of fog collectors with different structures [9-11]. **a**, schematic diagram of droplets transport in an inverted trapezoidal fog collector (Reproduced with permission from ref. 9). **b**, image transmission of droplet clusters on an inverted trapezoidal fog collector (Reproduced with permission from ref. 9). **c**, the influence of fog collector materials on water collection efficiency (Reproduced with permission from ref. 9). **d ~ f**, harp style fog collector (Reproduced with permission from ref. 10). **g**, comparison of water collection rates between harp style fog collectors and fog mesh (Reproduced with permission from ref. 10).

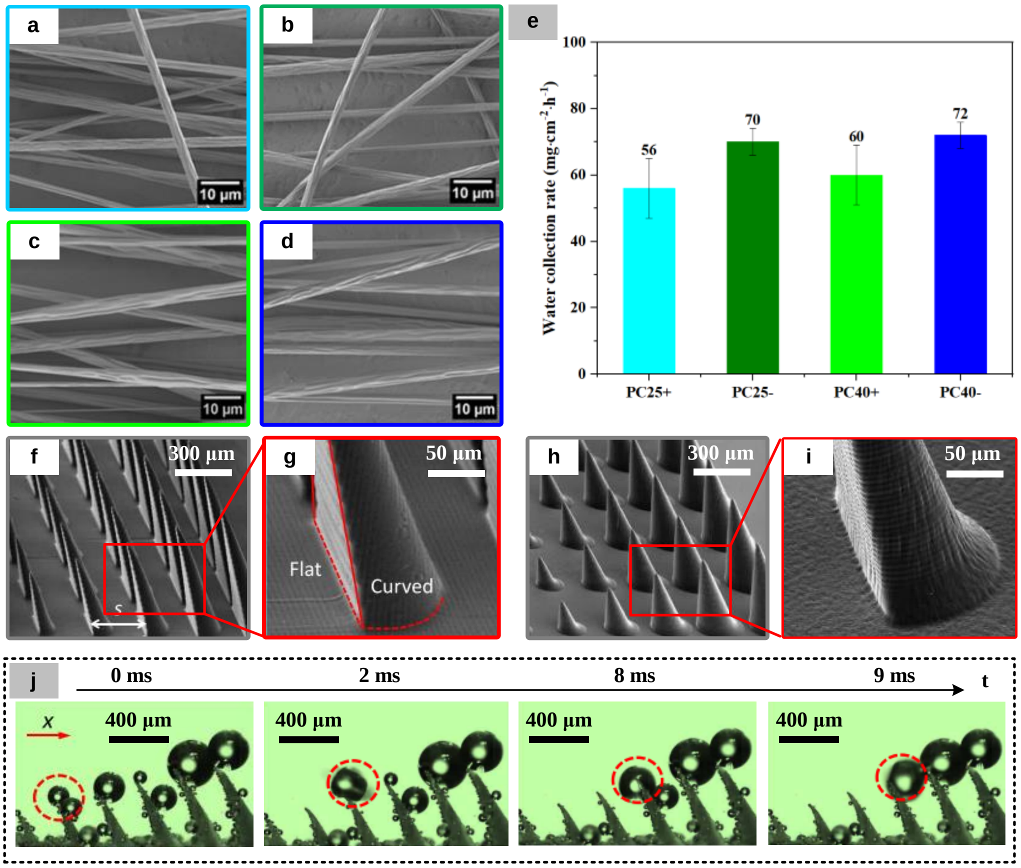

Fig. S5| Different directional transport droplet materials and water collection performance [12,13]. **a**, ~ **d**, electron microscopy images of polycarbonate fibers (PC) (Reproduced with permission from ref. 13). **e**, PC fog collection performance with different voltages and polarities (PC25+, PC25-, PC40+, PC40- are the conditions for the preparation of polycarbonate (PC) electrospun nets. PC – polycarbonate; 25 - temperature 25 ℃, relative humidity 25%, - – negative electrospinning polarity.) (Reproduced with permission from ref. 13). **f** ~ **i**, droplet-oriented transport materials prepared by 3D printing technology (Reproduced with permission from ref. 14). **j**, images of the position of droplets on the surface of transported materials at different times (Reproduced with permission from ref. 14).

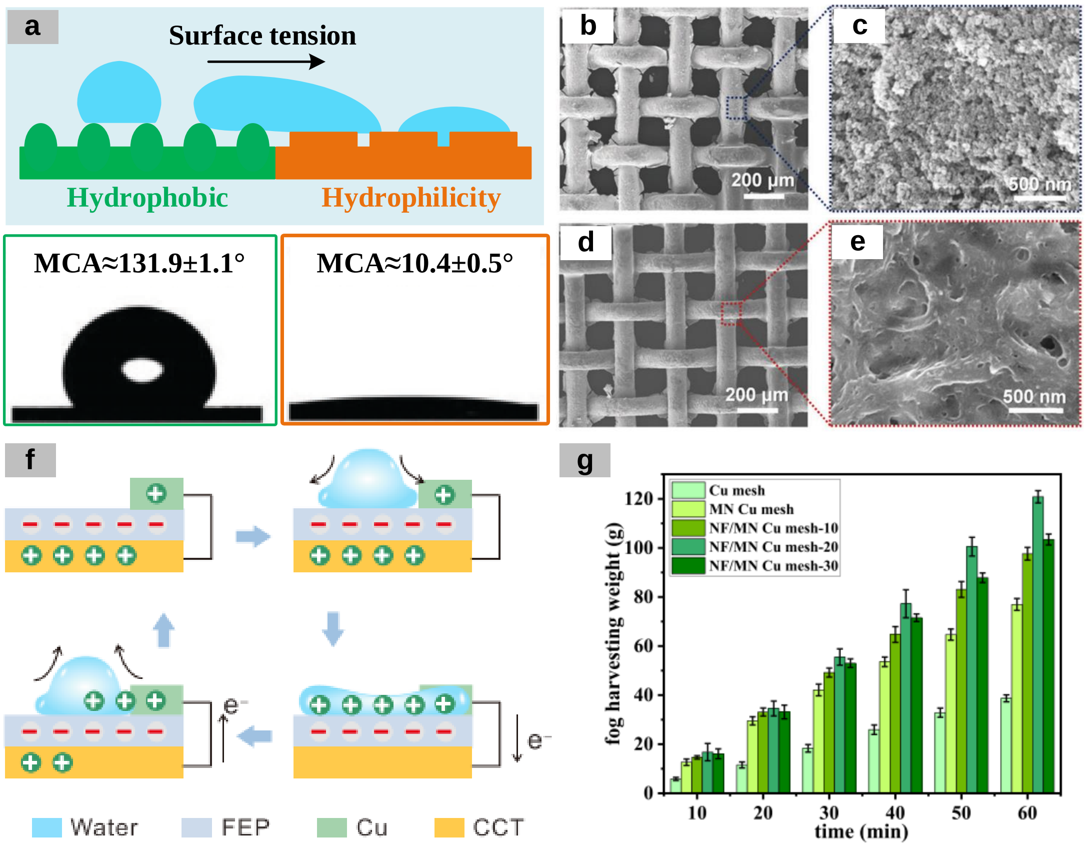

Fig. S6| Principle and fog collection performance of Janus fog collector [15-17]**. a**, schematic diagram of the basic principle of Janus structure for transporting liquid droplets (MCA - droplet contact angle for mixtures) (Reproduced with permission from ref. 18). **b** ~ **e**, electron microscopy images of Janus fog mesh (Reproduced with permission from ref. 16). **f**, Janus structure formed by utilizing charges (FEP - fluorinated ethylene propylene; CCT - conductive cloth tape) (Reproduced with permission from ref. 17). **g**, fog collection performance of Janus fog collectors made of different materials (NF - nanofiber; MN - micro-needle) (Reproduced with permission from ref. 15).

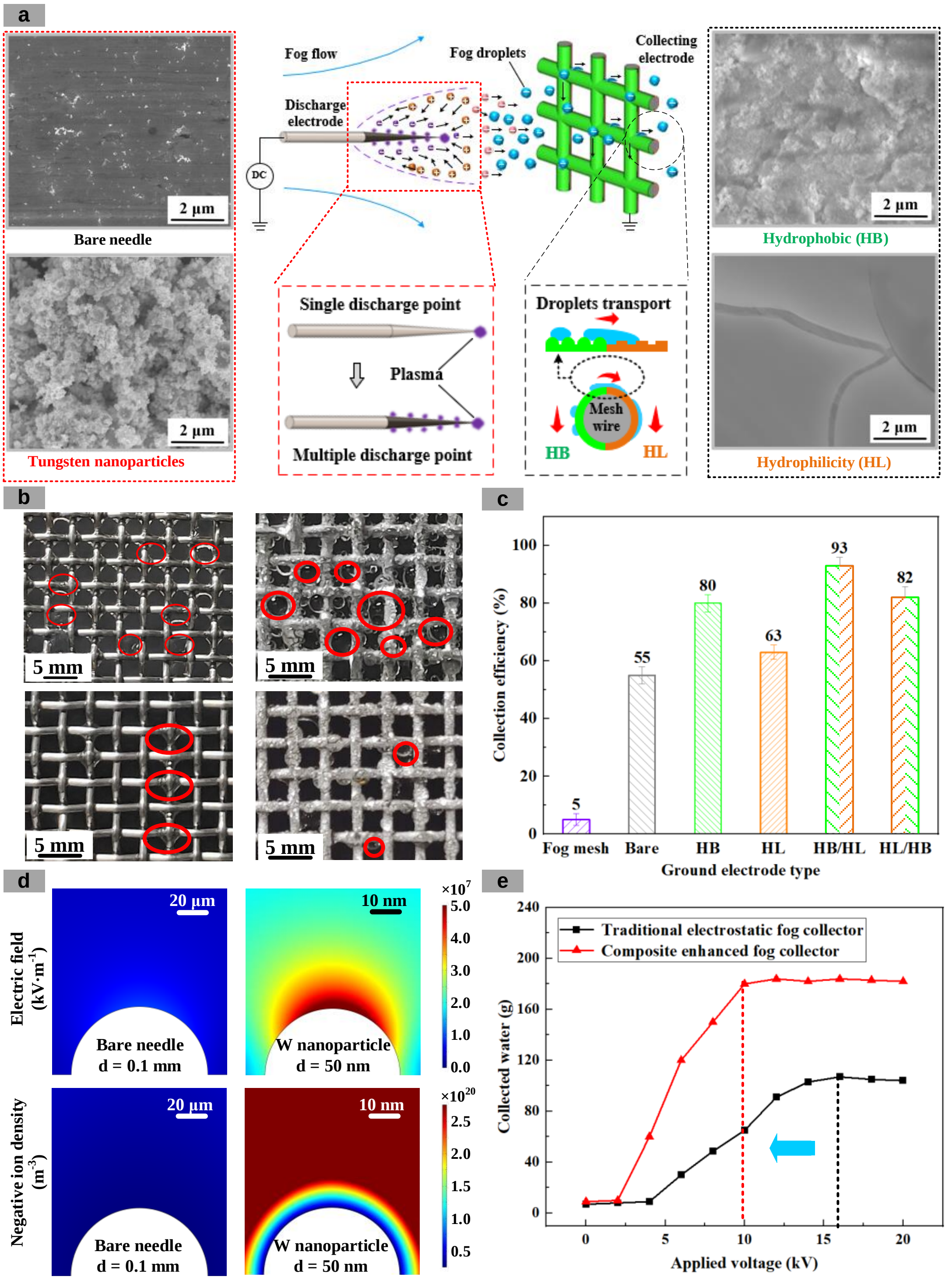

Fig. S7| Method for enhancing the collection efficiency of EFC by composite nanomaterials (Reproduced with permission from ref. 19)**. a**, the basic principle of composite efficiency enhancing EFC. **b**, different collection electrode droplet condensation images. **c**, the fog collection efficiency of EFC with different collection electrodes. **d**, the distribution of electric field and ion concentration near the discharge

of metal nanoparticles and traditional electrode tips. **e**, comparison of performance between composite enhanced EFC and traditional EFC.

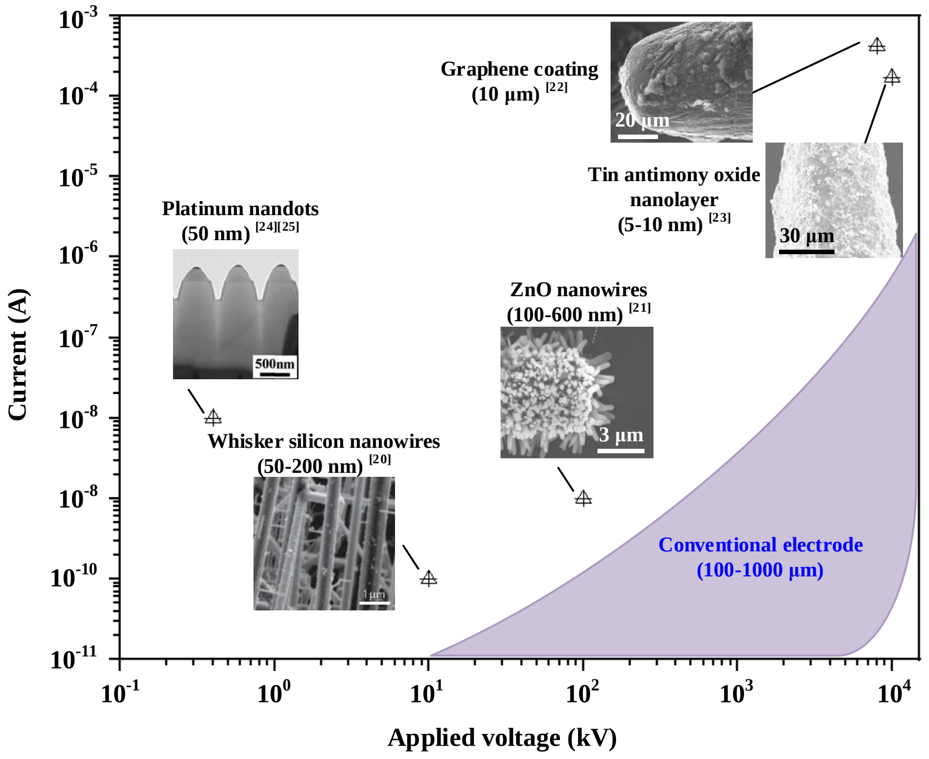

Fig. S8| Comparison of discharge currents between different micro/nano electrodes and traditional electrodes (Reproduced with permission from ref. 20-25)**.**

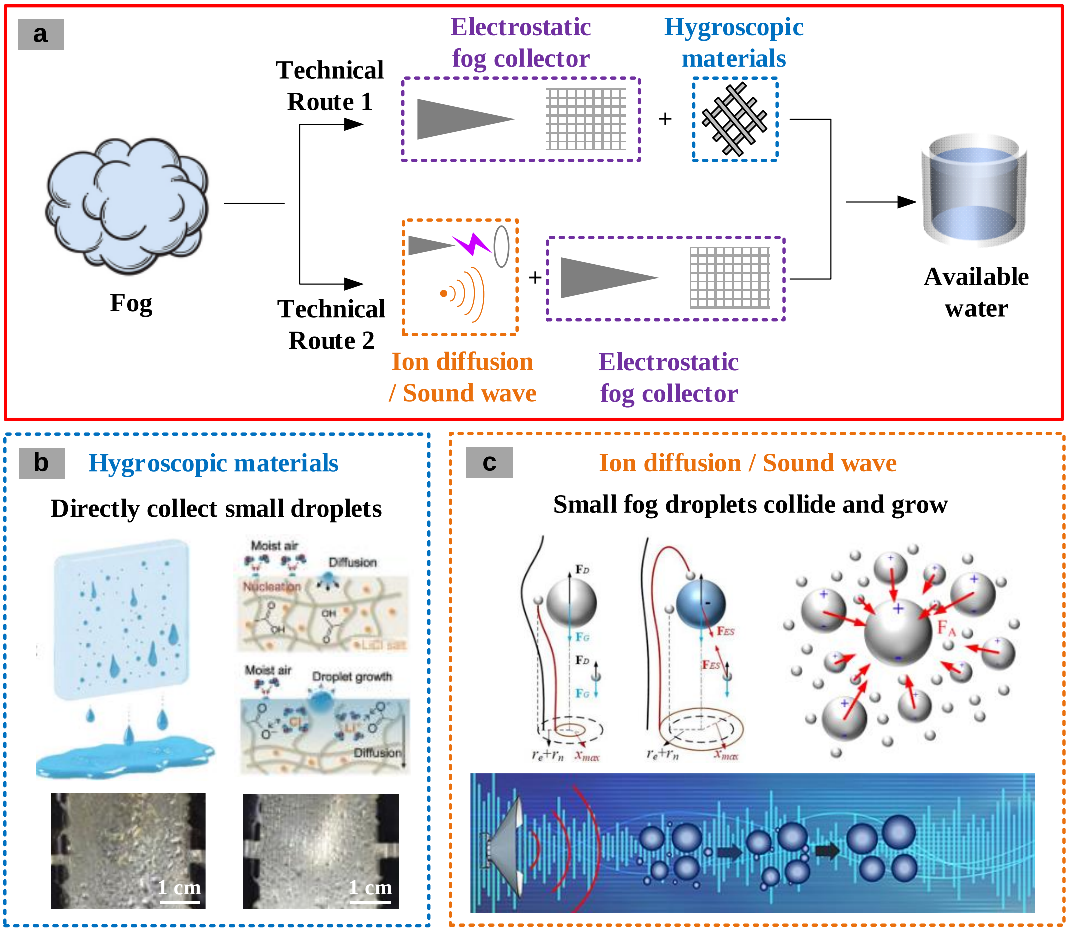

Fig. S9| Expanding the particle size range of EFC applied droplets to improve fog collection efficiency (Reproduced with permission from ref. 26-30). **a**, two technical routes to expand the particle size range of EFCs for collecting fog droplets. **b**, hygroscopic materials. **c**, electric charges or sound waves promote the collision and growth of droplets (F_D-fluid drag force; F_G-gravity force; F_{ES}-electric field force; F_A-dielectrophoretic force).

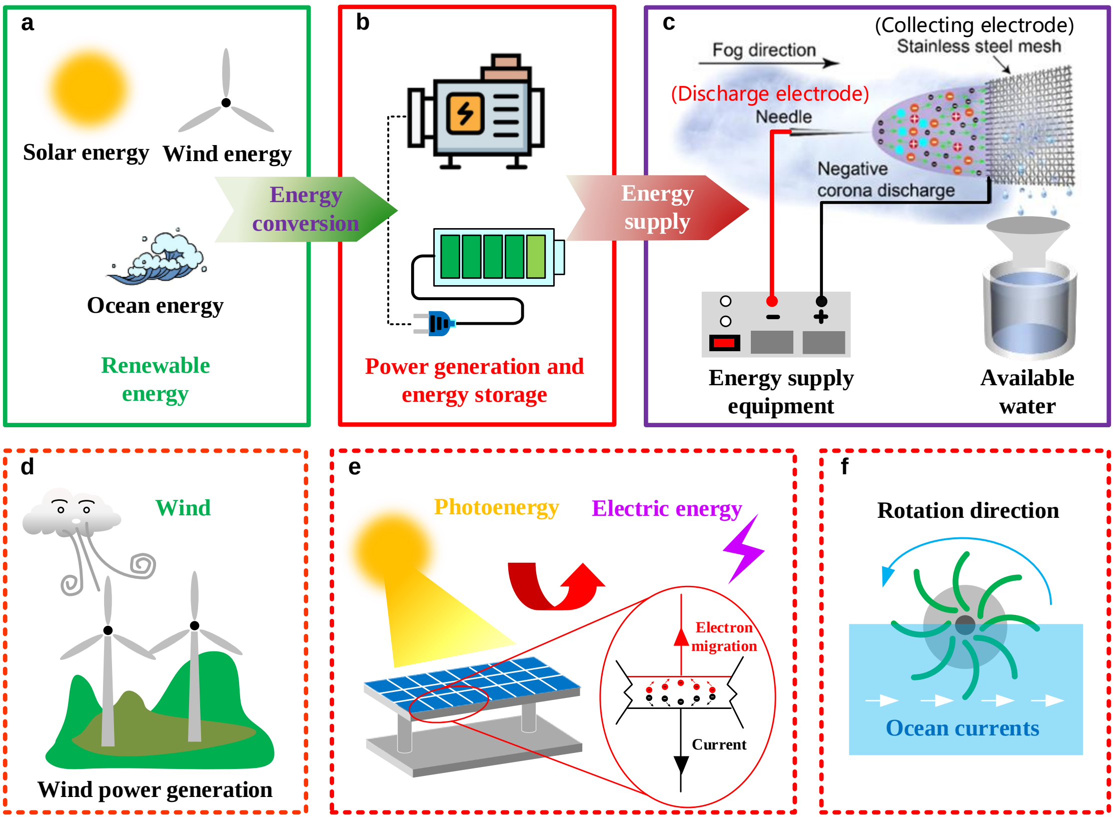

Fig. S10| Development diagram of EFC's self-powered system. a, three renewable energy sources in mountainous and island areas. **b**, power generation and energy storage system. **c)** EFCs (Reproduced with permission from ref. 31). **d**, wind power generation system. e, solar power generation system. f, ocean power generation system.

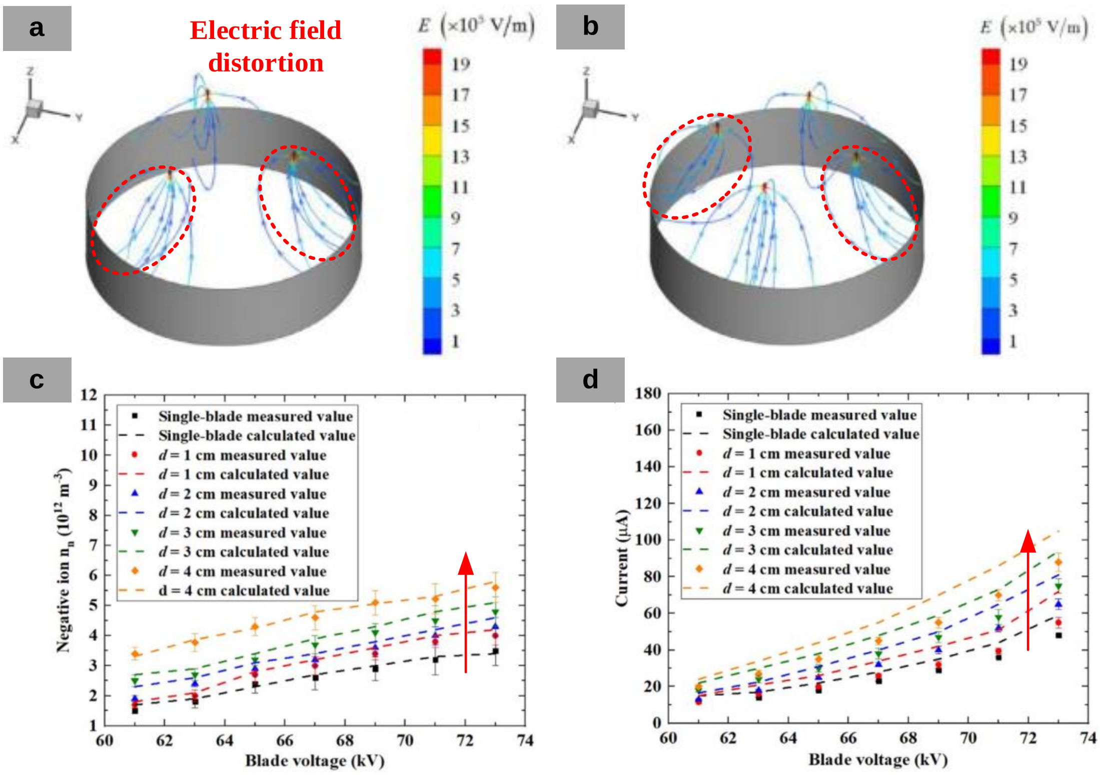

Fig. S11| Interaction diagram between discharge points during gas discharge with array electrodes [32,33]. **a**, electric field distribution of three needle electrodes (Reproduced with permission from ref. 33). **b**, electric field distribution of four needle electrodes (Reproduced with permission from ref. 33). **c**, the concentration of negative ions generated by single needle electrodes and double needle electrodes (n_n – negative ion concentration; *d* - distance between electrodes) (Reproduced with permission from ref. 32). **d**, the corona current generated by single needle electrode and double needle electrode (Reproduced with permission from ref. 32).

References for Supplementary information

1 Zhang, J., Kong, L., Qu, J., Wang, S. & Qu, Z. Numerical and experimental investigation on configuration optimization of the large-size ionic wind pump. *Energy* **171**, 624-630 (2019).

2 Feng, J., Wang, C., Liu, Q. & Wu, C. Enhancement of heat transfer via corona discharge by using needle-mesh and needle-fin electrodes. *Int. J. Heat Mass Transfer* **130**, 640-649 (2019).

3 Wang, Y. *et al.* Insights into the role of ionic wind in honeycomb electrostatic precipitators. *J. Aerosol Sci.* **133**, 83-95 (2019).

4 Zhang, M. *et al.* Enhancing fog collection by optimizing wettability combination and fork-row collector arrangement: light and heavy fog. *J. Phys. D: Appl. Phys.* **56**, 495204 (2023).

5 Zhang, M. *et al.* Numerical simulation of the enhancing effect of micro−nano protrusions on electrostatic fog harvesting. *J. Phys. D: Appl. Phys.* **56**, 385201 (2023).

6 Yan, X. & Jiang, Y. Numerical evaluation of the fog collection potential of electrostatically enhanced fog collector. *Atmos. Res.* **248**, 105251 (2021).

7 Jiang, Y., Xu, R., Liu, S., Liu, G. & Yan, X. Electrostatic fog collection mechanism and design of an electrostatic fog collector with nearly perfect fog collection efficiency. *Chem. Eng. Sci.* **247**, 117034 (2022).

8 Li, D. *et al.* Efficient direction-independent fog harvesting using a corona discharge device with a multi-electrode structure. *Plasma Sci. Technol.* **24**, 095502 (2022).

9 Rajaram, M., Heng, X., Oza, M. & Luo, C. Enhancement of fog-collection efficiency of a raschel mesh using surface coatings and local geometric changes. *Colloids Surf. A: Physicochem. Eng. Aspects* **508**, 218-229 (2016).

10 Shi, W. *et al.* Harps under heavy fog conditions: superior to meshes but prone to tangling. *ACS Appl. Mater. Interfaces* **12**, 48124-48132 (2020).

11 Shi, W., van der Sloot, T. W., Hart, B. J., Kennedy, B. S. & Boreyko, J. B. Harps enable water harvesting under light fog conditions. *Adv. Sust. Syst.* **4**, 2000040 (2020).

12 Li, J., Li, W., Han, X. & Wang, L. Sandwiched nets for efficient direction-independent fog collection. *J. Colloid Interface Sci.* **581**, 545-551 (2021).

13 Ura, D. P. *et al.* Surface potential driven water harvesting from fog. *ACS Nano* **15**, 8848-8859 (2021).

14 Shile, F. *et al.* Tip-induced flipping of droplets on Janus pillars: From local reconfiguration to global transport. *Sci. Adv.* **6**, eabb4540 (2020).

15 Li, H. *et al.* A quadruple biomimetic hydrophilic / hydrophobic Janus composite material integrating Cu(OH)2 micro-needles and embedded bead-on-string nanofiber membrane for efficient fog harvesting. *Chem. Eng. J.* **455**, 140863 (2023).

16 Chen, J., Liu, Y., Guo, D., Cao, M. & Jiang, L. Under-water unidirectional air penetration via a Janus mesh. *Chem. Commun.* **51**, 11872-11875 (2015).

17 Zhang, S. *et al.* Bioinspired Asymmetric Amphiphilic Surface for Triboelectric Enhanced Efficient Water Harvesting. *Nat. Commun.* **13**, 4168 (2022).

18 Deng, Q. *et al.* One droplet toward efficient alcohol detection using femtosecond laser textured micro/nanostructured surface with superwettability. *Small Meth.* **7**, 2300290 (2023).

19 Li, D. *et al.* Advanced fog harvesting method by coupling plasma and micro/nano materials. *ACS Appl. Mater. Interfaces* **16**, 10984-10995 (2024).

20 Banan Sadeghian, R. & Islam, M. S. Ultralow-voltage field-ionization discharge on whiskered silicon nanowires for gas-sensing applications. *Nat. Mater.* **10**, 135-140 (2011).

21 Yang, W., Zhu, R. & Zong, X. ZnO nanowire-based corona discharge devices operated under hundreds of volts. *Nanoscale Res. Lett.* **11**, 90 (2016).

22 Wu, Y. *et al.* Greener corona discharge for enhanced wind generation with a simple dip-coated carbon nanotube decoration. *J. Phys. D: Appl. Phys.* **50**, 395304 (2017).

23 Wang, J., Zhu, T., Cai, Y., Bao, Y. & Wang, J. Comparison of the generation characteristics and application performance of nanomaterials-enhanced ionic wind. *Int. Commun. Heat Mass Transfer*

117, 104734 (2020).

24 Liu, H. *et al.* Self-organization of a hybrid nanostructure consisting of a nanoneedle and nanodot. *Small* **8**, 2807-2811 (2012).

25 Liu, H., Yadian, B., Liu, Q., Gan, C. L. & Huang, Y. A hybrid nanostructure array for gas sensing with ultralow field ionization voltage. *Nanotechnol.* **24**, 175301 (2013).

26 Li, J. *et al.* Numerical analysis of collision characteristics between charged drop and neutral droplet under uniform electric field. *J. Phys. D: Appl. Phys.* **54**, 455201 (2021).

27 Zhang, M. *et al.* Investigation of the effects of parallel electric field on fog dissipation. *J. Phys. D: Appl. Phys.* **56**, 375204 (2023).

28 Yang, Y. *et al.* Agglomeration of oil droplets assisted by low-frequency sonic pretreatment. *Powder Technol.* **428**, 118860 (2023).

29 Nah, S. H. *et al.* Moisture Absorbing and Water Self‐Releasing from Hybrid Hydrogel Desiccants. *Adv. Funct. Mater.* **34**, 2313881 (2023).

30 Yang, Y., Chen, H., Li, C. & Wang, P. Ion induced nucleation of charged droplets enhanced by external electric field. *Phys. Plasmas* **31**, 073505 (2024).

31 Gu, G. *et al.* A water collection system with ultra-high harvest rate and ultra-low energy consumption by integrating triboelectric plasma. *Nano Energy* **96**, 107081 (2022).

32 Li, D. *et al.* Study on the Interaction Mechanism of Double-blade Corona Discharge with a Large Discharge Gap. *Plasma Sc. Technol.* **25**, 045404 (2023).

33 Qu, J., Zhang, J., Li, M. & Tao, W. Heat dissipation of electronic components by ionic wind from multi-needle electrodes discharge: experimental and multi-physical analysis. *Int. J. Heat Mass Transfer* **163**, 120406 (2020).
